# *Phytophthora*: an ancient, historic, biologically and structurally cohesive and evolutionarily successful generic concept in need of preservation

**DOI:** 10.1186/s43008-022-00097-z

**Published:** 2022-06-27

**Authors:** Clive Brasier, Bruno Scanu, David Cooke, Thomas Jung

**Affiliations:** 1 Forest Research, Alice Holt Lodge, Farnham, Surrey, GU10 4LH UK; 2 Department of Agricultural Sciences, University of Sassari, Viale Italia 39A, 07100 Sassari, Italy; 3 The James Hutton Institute, Invergowrie, Dundee, DD2 5DA UK; 4 Department of Forest Protection and Wildlife Management, Phytophthora Research Centre, Mendel University in Brno, 613 00 Brno, Czech Republic; 5 Phytophthora Research and Consultancy, 83131 Nussdorf, Germany

**Keywords:** Oomycetes, Downy mildews, Economic impact, Molecular phylogeny, Paraphyly, Cladism, Biosecurity

## Abstract

The considerable economic and social impact of the oomycete genus *Phytophthora* is well known. In response to evidence that all downy mildews (DMs) reside phylogenetically within *Phytophthora*, rendering *Phytophthora* paraphyletic, a proposal has been made to split the genus into multiple new genera. We have reviewed the status of the genus and its relationship to the DMs. Despite a substantial increase in the number of described species and improvements in molecular phylogeny the *Phytophthora* clade structure has remained stable since first demonstrated in 2000. Currently some 200 species are distributed across twelve major clades in a relatively tight monophyletic cluster. In our assessment of 196 species for twenty morphological and behavioural criteria the clades show good biological cohesion. Saprotrophy, necrotrophy and hemi-biotrophy of woody and non-woody roots, stems and foliage occurs across the clades. Phylogenetically less related clades often show strong phenotypic and behavioural similarities and no one clade or group of clades shows the synapomorphies that might justify a unique generic status. We propose the clades arose from the migration and worldwide radiation ~ 140 Mya (million years ago) of an ancestral Gondwanan *Phytophthora* population, resulting in geographic isolation and clade divergence through drift on the diverging continents combined with adaptation to local hosts, climatic zones and habitats. The extraordinary flexibility of the genus may account for its global ‘success’. The 20 genera of the obligately biotrophic, angiosperm-foliage specialised DMs evolved from *Phytophthora* at least twice via convergent evolution, making the DMs as a group polyphyletic and *Phytophthora* paraphyletic in cladistic terms. The long phylogenetic branches of the DMs indicate this occurred rather rapidly, via paraphyletic evolutionary ‘jumps’. Such paraphyly is common in successful organisms. The proposal to divide *Phytophthora* appears more a device to address the issue of the convergent evolution of the DMs than the structure of *Phytophthora *per se. We consider it non-Darwinian, putting the emphasis on the emergent groups (the DMs) rather than the progenitor (*Phytophthora*) and ignoring the evolutionary processes that gave rise to the divergence. Further, the generic concept currently applied to the DMs is narrower than that between some closely related *Phytophthora* species. Considering the biological and structural cohesion of *Phytophthora*, its historic and social impacts and its importance in scientific communication and biosecurity protocol, we recommend that the current broad generic concept is retained by the scientific community.

**Supplementary Information:**

The online version contains supplementary material available at 10.1186/s43008-022-00097-z.

*Taxonomy’s purpose is to foster clear scientific communication and the job of taxonomists is to refine it with that in mind. In doing so, Taxonomists must not only recommend improved communication going forward, but also weigh the costs of altering longstanding, effective communication* (Booth 1978)

## INTRODUCTION

The era of molecular phylogeny has provided strong evidence that the downy mildews (DMs) are as a group polyphyletic, having evolved at least twice from *Phytophthora* ancestors (Cooke et al. 2000; Runge et al. 2011; Jung et al. 2017a; Bourret et al. 2018; Scanu et al. 2021). This has led to a proposal to distribute the main phylogenetic clades of *Phytophthora* among several new genera (Runge et al. 2011), further indicated recently by Crous et al. (2021a). To assess the merits of this proposal we review here the environmental, economic and social impact, and the biological and phylogenetic characteristics of the genus, including its relationship to the DMs. We conclude that the case for retaining *Phytophthora* as a single genus is overwhelming.

## COMMENTARY

### Historical background

*Phytophthora* is arguably the world’s most historic and economically significant genus in plant pathology. A comprehensive timeline of milestones for the genus is given in Table 1. In current classifications *Phytophthora* is usually assigned to the phylum *Oomycota*, which in turn are widely accepted as belonging to the heterokont algal-derived, but still somewhat debated, *Straminipila* within the kingdom *Chromista* (Dick 2001; Beakes et al. 2012). Together with other oomycetes, Phytophthoras are diploid with gametangial meiosis (Sansome 1961, 1965) and a genetic system akin to that of vascular plants (Brasier 1992; Goodwin 1997). They form indeterminate sporangiophores bearing alga-like sporangia that, in turn, release flagellate zoospores; and alga-like sexual oogonia and antheridia. Like most oomycetes, Phytophthoras exhibit a strong dependence on free water or high humidity for sporangial formation, zoospore spread and infection.

**Table 1:** Timeline of biological milestones in the genus *Phytophthora*

Year	Milestones in the genus *Phytophthora*	References
1845	Potato blight epidemic in Europe	Bourke (1991)
1876	*Phytophthora infestans* designated cause of potato blight	de Bary (1876)
1892	*Phytophthora* assigned to *Peronosporales*	Fischer (1892)
1922	‘Heterothallism’ discovered (*P. faberi*; syn. *P. palmivora*)	Ashby (1922), Gadd (1924)
1925	First genus-wide physiological studies in *Phytophthora*	Leonian (1925)
1931	First major taxonomic treatises on *Phytophthora*	Tucker (1931), Leonian (1934)
1935	Thiamin requirement for growth demonstrated	Ronsdorf (1935), Leonian and Lilly (1938)
1952	Mode of evolution from lower to higher Peronosporales proposed	Gäumann and Wynd (1952)
1960	A1 and A2 compatibility types in ‘heterothallics’ are bisexual	Galindo and Gallegly (1960)
1963	Taxonomic key to ~ 40 known *Phytophthora* species	Waterhouse (1963)
1963	Oomycetes (*Achlya*, *Pythium, Phytophthora, Sclerospora*) shown to be diploid with gametangial meiosis	Sansome (1961, 1963, 1965, 1966)
1964	Exogenous sterols required for sexual reproduction	Elliot et al. (1964), Haskins et al. (1964), Hendrix (1964), Leal et al. (1964)
1972	‘Battle for or against diploidy’ convention in Bari, Italy	Brasier (2008)
1972	Chemical/hormonal induction of sexual differentiation, including selfing, in A1 x A2 interactions	Brasier (1972), Ko (1978)
1973	*Phytophthora infestans* shown to be diploid. First report of chromosomal structural hybrids (reciprocal translocation heterozygotes) in A1 x A2 outcrossing Phytophthoras	Sansome and Brasier (1973)
1980	Mitiotic segregation of the homozygous from the heterozygous mating type suppressed by reciprocal translocation heterozygosity	Sansome (1980)
1980	First molecular taxonomy based on protein and DNA polymorphisms	Kaosiri and Zentmyer (1980), Erselius and Shaw (1982), Hansen et al. (1986), Förster et al. (1988, 1990)
1989	Oomycetes assigned to *Straminipila*	Patterson (1989), Dick (2001)
1990	Designation of *Halophytophthora* gen. nov	Ho and Jong (1990)
1996	*Phytophthora* reaches ~ 58 described species	Erwin and Ribeiro (1996)
1998	First interspecific hybrid described	Man in’t Veldt et al. (1998)
1997	Role of effector molecules in *Phytophthora* host specificity and pathogenesis	Kamoun et al. (1997)
2000	First molecular phylogeny of the oomycetes. Major *Phytophthora* clades identified. Downy mildews (*Peronospora*, *Bremia*) shown as evolved from *Phytophthora*	Cooke et al. (2000)
2002	World-wide surveys reveal many new Phytophthoras undetected in natural ecosystems	Jung et al. (2002, 2017b, 2018b, 2020, 2021; d), Brasier et al. (2010), Burgess et al. (2018), Dang et al. (2021)
2004	First multigene phylogeny of *Phytophthora*	Kroon et al. (2004)
2007	First multigene phylogeny of the oomycetes. Clade structure sustained	Göker et al. (2007)
2009	400–600 *Phytophthora* species predicted	Brasier (2009)
2014	Divergence of *Phytophthora* Clades predicted at 19.8–39 m years ago	Matari and Blair (2014)
2017	Designation of *Nothophytophthora* gen. nov. *Phytophthora* origin pre the Gondwana-Laurasia separation (> 180 Myr) proposed	Jung et al. (2017a)
2018	Multiple evolution of downy mildews from Phytophthoras demonstrated	Bourret et al. (2018)
2021	First phylogeny from genome-wide sequencing. Clade structure sustained	Van Poucke et al. (2021)
2021	*Phytophthora* reaches 200 described species	Scanu et al. (2021), Chen et al. (2022)

Within the oomycetes, *Phytophthora* is now assigned to the order *Peronosporales*, the vast majority of which are plant pathogens (Runge et al. 2011; Thines and Choi 2016; Jung et al. 2017a, 2018a). Amongst others this order includes the genera *Halophytophthora* and *Calycofera, Phytophthora*’s sister genus *Nothophytophthora*, and 20 genera of DMs including *Bremia*, *Peronospora, Plasmopara* and *Sclerospora* (cf. Thines and Choi 2016; Jung et al. 2017a; McCarthy and Fitzpatrick 2017; Bourret et al. 2018; Scanu et al. 2021; Maia et al. 2022)*. Phytophthora* and *Nothophytophthora* are mainly soil and water inhabiting, necrotrophic to hemibiotrophic pathogens forming zoosporic sporangia, whereas the DMs are aerial, obligate biotrophic pathogens with often conidia-like sporangia. The first DM genus, *Peronospora*, was erected by Corda (1837). The nomenclatural history of *Phytophthora* began with the potato blight epidemic in western Europe in the 1840s that led to the infamous Irish potato famine (Large 1940; Bourke 1991). The causal agent was initially named *Botrytis infestans* by Montagne (1845). It was then redesignated *Peronospora trifurcata* by Unger (1847), *Peronospora infestans* by Caspary in (1853) (published in Rabenhorst's *Herbarium vivum Mycologicum exsiccati* no*.* 1879), and finally renamed *Phytophthora infestans* by de Bary (1876), with *P. infestans* as the ‘type species’ for the new genus *Phytophthora* (Table 1).

With the expansion of plant pathology as a discipline in the early 1900s the number of described *Phytophthora* species gradually increased. Rosenbaum (1917), Tucker (1931) and Leonian (1934) produced the first morphologically based keys to meet the growing need for accurate communication. Tucker (1931) accepted twenty species and was notable in emphasising the value of sporangial and gametangial morphology and temperature-growth relations as taxonomic criteria (Brasier 1991). Waterhouse (1963) developed a key based on assigning around 40 species to six morphological groups, introducing a sense of cohesion to a rather loosely structured mass of information (Gallegly 1983). Later, Waterhouse (1970) listed 60 *Phytophthora* species with a Latin description and/or a designated type, but 19 of these were later discarded in the *Phytophthora* monograph of Erwin and Ribeiro (1996), who accepted 58 species; seven of which were later considered invalidly published or lost. The Waterhouse morphological system was developed further in the keys of Newhook et al. (1978) and Stamps et al. (1990). By the late 1980s, however, population-based, karyotype-based and molecular polymorphism-based systematic criteria were being advocated, heralding advancement towards a revised species concept and a more natural evolutionary phylogeny, including the likelihood that the *Phytophthora* genetic system was generating inter-specific hybrids (Brasier 1991; Hansen 1991).

### Rapid increase in described Phytophthora species

From around 2000 the number of described species increased rapidly. This was partly due to the unravelling of morphospecies complexes by combinations of classical and molecular methods (e.g. Brasier et al. 2003; Hansen et al. 2019; Jung et al. 2011; Bertier et al. 2013; Ginetti et al. 2014; Safaiefarhani et al. 2015; Weir et al. 2015); and partly to the discovery of many new species and infraspecific lineages during dedicated surveys in forests and natural ecosystems, especially remote regions with low accessibility (e.g. Jung et al. 2003, 2011, 2017b, c, d, 2018b, 2020, 2021; Rea et al. 2011; Reeser et al. 2011; Brasier et al. 2012; Scanu et al. 2015; Burgess et al. 2018; Dang et al. 2021).

Within a decade the number of formally described Phytophthoras had surpassed 100, and it was estimated that the number of extant *Phytophthora* species could be between 200 and 600 (Brasier 2009). Currently, the number of formally described and accepted taxa has reached 200 (Scanu et al. 2021; Chen et al. 2022) and many other new taxa have been designated informally (Brasier et al. 2003; Hüberli et al. 2013; Oh et al. 2013; Jung et al. 2017d, 2018b, 2020). Another 2–400 species may remain to be discovered in the world’s unsurveyed forests and natural ecosystems (Brasier 2009).

**Fig. 1:**
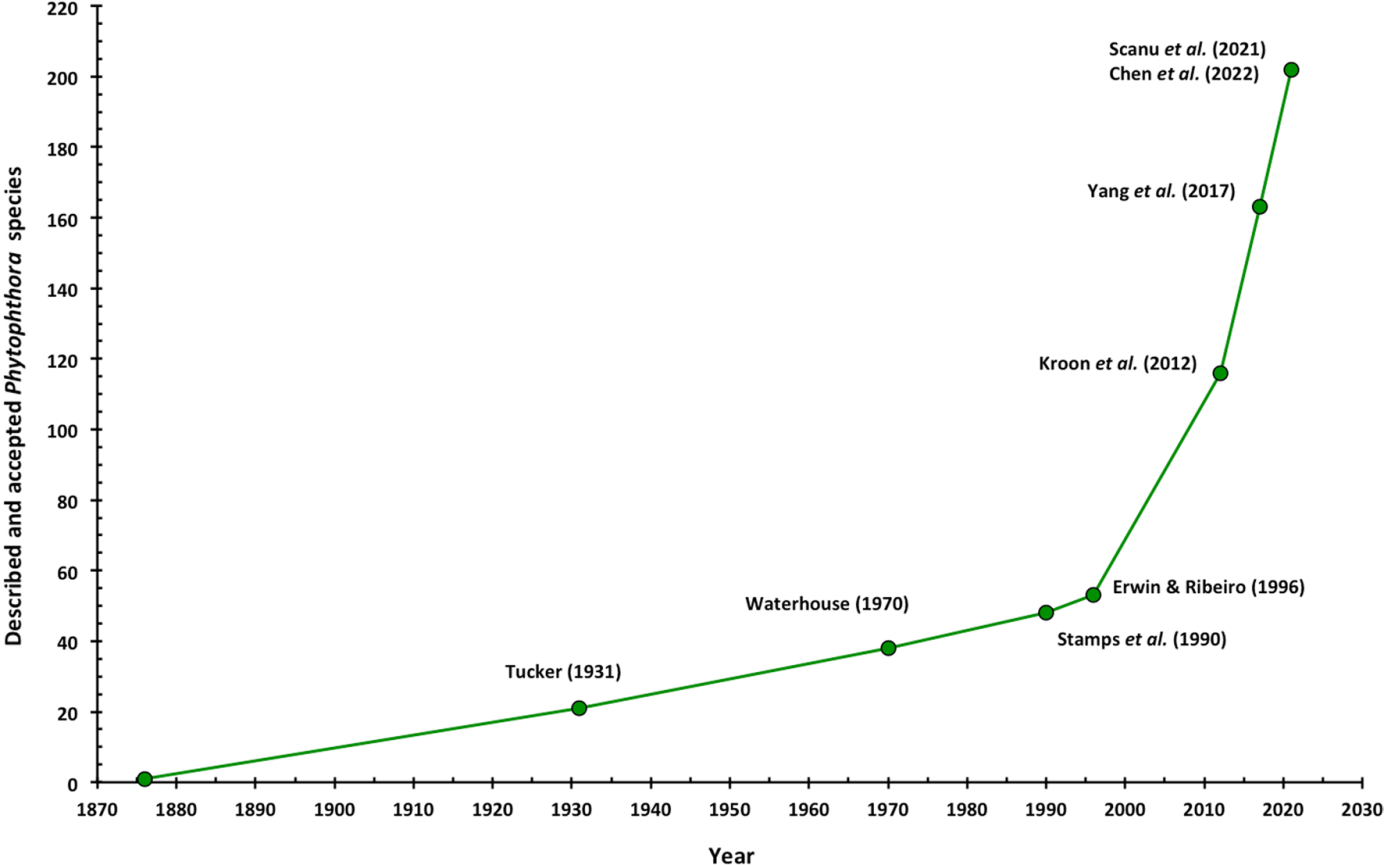
Number of described and accepted *Phytophthora* species over time. Adapted from Brasier (2009). Data from pre-molecular era publications (Tucker 1931; Waterhouse 1970; Stamps et al. 1990; Erwin and Ribeiro 1996) include only those species currently accepted as valid species

Figure 1 summarises the numerical chronology of described *Phytophthora* species, highlighting the exponential increase in species numbers over the past two decades.

### Economic, environmental and social impact of the genus

Having been born out of a disastrous famine in Western Europe the genus *Phytophthora* was imbued with a degree of notoriety from its inception. Any modern perception of the genus needs to be much broader, in part because a definition of ‘importance’ in solely human terms is an artificial, not a biological, construct: *P. infestans* is no more biologically significant in its natural environment than are most other Phytophthoras in theirs. Nonetheless the genus contains a remarkable number of individually infamous pathogens, including (in addition to *P. infestans*) *P. capsici*, *P. cinnamomi*, *P. megakarya, P. nicotianae*, *P. palmivora*, *P. plurivora* and *P. ramorum*. Overall, the anthropogenically-related impacts of Phytophthoras are enormous (Tables 2, 3, Additional files 1, 2: Tables S1, S2). Most of these impacts are driven by introductions to environments with highly susceptible hosts, use of crop monocultures, host stress due to ‘off-site’ cultivation and climate change, or a combination of these (e.g. Brasier and Scott 1994; Erwin and Ribeiro 1996; Jung 2009; Jung et al. 2000, 2018a; Rizzo et al. 2002; Shearer et al. 2004; Brasier and Webber 2010; Lamour 2013).

**Table 2:** Examples of the ecological, economic, social and scientific impacts of selected *Phytophthora* species

*Phytophthora* species^a^	Clade	First described	Environments	Main diseases caused and impacts	Scopus indexed articles and their citations
*P. agathidicida*	5	2015	Forest, park	Dieback of Kauri, one of the world’s largest and longest-living conifer species, in New Zealand, spreading since 1974. Negative impact on both forest ecosystems and Mauri society due to the ecological and cultural significance of Kauri trees	29/171
*P. austrocedri* (syn. *P. austrocedrae*)	8	2005	Forest, natural ecosystem	Dieback and mortality of native *Austrocedrus* forests in the southern Andes. Dieback and mortality of native Juniper, UK, initially associated with restoration planting of infested nursery stock	26/135^b^
*P. cactorum*	1	1886	Agriculture, forest, nursery	Root, collar, crown and fruit rots and stem cankers on over 200 species of trees, ornamentals, and fruit crops in 160 genera worldwide	586/6481
*P. capsici*	2	1922	Horticulture	Phytophthora blight of *Capsicum* in the Americas and Southeast Asia, and a major limiting factor to vegetable production globally, especially cucurbits, tomatoes, and succulent beans, causing up to 100% losses in individual fields	1559/18,150
*P. cinnamomi*	7	1922	Forest, heathland, nursery, garden	Dieback of eucalypt forests and woodlands and mass destruction of World Heritage heath flora in Western Australia since 1950s. Heavy mortality of Fagaceae in forests of southeastern US since 1940s and southern Europe since ~ 1990s. Damage to ornamental nursery trade in Europe since 1970s. Listed as one of the 100 worst invasive alien species; pathogenic to ~ 5000 trees, woody ornamentals, and herbaceous plants worldwide	1331/12,976
*P. cryptogea*	8	1919	Horticulture, nursery, garden	Root and collar rot on a wide range of crops, fruit trees and ornamentals worldwide. Particularly important pathogen in greenhouses	272/5261
*P. fragariae*	7	1940	Horticulture	Red core root disease of strawberry since 1920s, causing serious economic losses in strawberry plantations across humid regions of Europe and North America, with severely reduced yields and small poor-quality fruit. In Canada production losses to growers of Can$ 1500 per ha	178/2091
*P. infestans*	1	1876	Agriculture, horticulture	Late blight of potato and tomato, notorious for the Irish potato famine 1845–1849 resulting in mass starvation and migration. Currently still a serious threat to global food security worldwide, with US$ 6.7 million annually in yield losses and control costs	4241/44,346
*P. kernoviae*	10	2005	Forest, heathland, horticulture, park	Aerial bleeding cankers on European beech and leaf and shoot blights of *Rhododendron, Magnolia* spp., and wild bilberry in the UK and Ireland	59/976
*P. lateralis*	8	1942	Forest, nursery, park, shelterbelt	Root disease causing heavy mortality of Port Orford cedar (*Chamaecyparis lawsoniana*) in its native range in Oregon and California since 1950s. Serious impact on trade in this valuable commercially harvested timber. Recently spread to ornamental *C. lawsoniana* in western Europe. Social impacts through loss of business in nursery and forestry sectors	62/1471
*P. megakarya*	4	1979	Agroforestry	Main cause of Black pod disease of cocoa trees in central west Africa since the early 1900s, recently spread to Ghana. Loss of yield often > 30% for the economically important cocoa industry, worth ca US$ 70 billion annually	106/2301
*P. nicotianae* (syn. *P. parasitica*)	1	1896	Agriculture, horticulture, nursery, garden	Severe diseases of agricultural and horticultural crops worldwide, including foot rot and gummosis of citrus, black shank of tobacco and collar rot of tomato. Also on ornamentals. Broad host range, infects > 255 genera in 90 plant families	1235/17,464^b^
*P. palmivora*	4	1919	Agroforestry, nursery, garden	Major impact on the production of tropical tree crops including black stripe disease of rubber in Southeast Asia since early 1900s. Also Black pod disease of cocoa in Southeast Asia and the Caribbean, with annual global losses to the cocoa industry of ca 450,000 t valued at > US$ 1 billion. Many ornamental hosts	567/5892
*P. plurivora*	2	2009	Forest, nursery, park, garden	Root and collar rot and aerial stem cankers on a wide range of woody hosts in Europe and North America; involved in the decline of oak and beech across Europe. Severe impact on the ornamental nursery industry	66/644
*P. quercina*	12	1999	Forest, park	Host-specific fine root pathogen. A main driver of the chronic decline of oak forests across Europe, interacting with climatic extremes	53/896
*P. ramorum*	8	2001	Forest, nursery, garden	Over 200 plant hosts. High impact. Cause of Sudden oak death (native tanoak and other species) in the Western US since ~ 2000. Through loss of tanoak seed production, a significant impact on local wildlife and native American culture. Cause of Sudden larch death in the UK and Ireland since ~ 2010 with ~ 200 km^2^ plantation larch affected and millions felled Currently a threat to commercial timber production in the US (> US$ 30 billion) and the UK. Also damaging to the ornamental nursery trade in Europe and North America e.g. the rhododendron export trade in Canada (around US$ 5 million)	627/8560
*P. rubi* (syn. *P. fragariae* var. *rubi*)	7	2007	Horticulture	Extremely serious disease of raspberry plantations in Europe, North America, and elsewhere. EPPO A2 list, recommended for phytosanitary treatments	57/475^b^
*P. sojae* (syn. *P. megasperma* var. *sojae*)	7	1958	Horticulture	Devastating root and stem rot of soybean in the US, with an annual cost worldwide of US$ 1–2 billion	810/13,592^b^
*P. syringae*	8	1909	Horticulture, nursery, garden	Root and collar rot, stem cankers, leaf and shoot blights and fruit rot on a medium-wide range of host plants including fruit trees and lilac	71/2207
*P.* ×*alni*	7	2004	Riparian forest, nursery	Extensive mortality of riparian alder across Europe since 1990s, driven by planting of infested nursery stock. Impacts ecosystem functions and services and riverbank stability. EPPO alert list 1996 to 2001	72/772
*P.* ×*cambivora*	7	1927	Forest, horticulture, nursery, garden	Root and collar infections (Ink disease) of sweet chestnut and beech in Europe. Root rot of various fruit trees in Europe and the US since 1900s. Significant impact on ornamental nurseries	142/2259

^a^Associated references are shown in full in Additional file 1: Table S1

^b ^Data include species synonyms

**Table 3:** Examples of the ecological, economic and social impacts of disease syndromes or processes involving multiple *Phytophthora* species

Syndrome or process and location	Environments	No. of *Phytophthora* taxa and hybrids involved^a^	Clades	Impacts
Cocoa black pod disease: West Africa, Caribbean, South America, Southeast Asia	Plantation	5	2, 4, 5	Cocoa pod lesions. Heavy crop losses (cf. *P. megakarya*, Table 2). Impact on small scale local farming communities and on global chocolate industry
Oak decline: across Europe	Forest, park	26	1, 2, 3, 5, 6, 7, 8, 10, 12	Root lesions and sometimes also collar lesion leading to forest declines driven in part by introduced pathogens and interaction with climate change. Impact on forestry and recreation
Beech decline: across Europe	Forest, park	16	1, 2, 3, 5, 6, 7, 10, 12	Root lesions, collar lesions, stem lesions leading to forest declines driven in part by introduced pathogens and interaction with climate change. Impact on forestry and recreation
Dieback of Mediterranean maquis vegetation: La Maddelena archipelago, Italy	Natural vegetation	9	6, 7, 8	Root lesions, collar lesions, stem lesions leading to mortality and decline of natural vegetation in a National Park. Impact on tourism, biodiversity and natural heritage
Restoration plantings in native Mediterranean heath vegetation and woodlands: Bay area, California	Planting, specialist nursery	51	1, 2, 4, 6,7,8	Strong evidence for spread to native plant habitats of at least five *Phytophthora* species causing root lesions, collar lesions, dieback and mortality of the vegetation. Impact on biodiversity and natural heritage
Dieback of eucalypt forests, Banksia woodlands and heath vegetation: across Western Australia	Forest, natural vegetation	26		Root and collar rot resulting in devastating dieback of whole ecosystems. Many of the *Phytophthora* species involved are considered native; however, the most aggressive species with the widest host ranges are introduced invasives (*P. cinnamomi*, *P. elongata* and *P. multivora*). Impacts on biodiversity, conservation, forestry and natural heritage
Woody plant nurseries and outplantings: across Europe	Nursery, outplantings	65	1, 2, 3, 4, 6, 7, 8, 9, 12	Most of these *Phytophthora* taxa are not native to Europe but are now established in the wider environment causing diseases of trees and shrubs in forests and natural ecosystems (e.g. *P. austrocedri*, *P. cactorum*, *P. cinnamomi*, *P. kernoviae, P. multivora*, *P. plurivora*, *P. ramorum, P.* ×*alni*, *P.* ×*cambivora*). Direct impact on nurseries; indirect impact on forestry, private garden owners, recreation and natural heritage

^a^Lists of the individual taxa involved in each syndrome (including described species and currently informally designated species) and the full citations of the associated references are shown in Additional file 2: Table S2

**Fig. 2:**
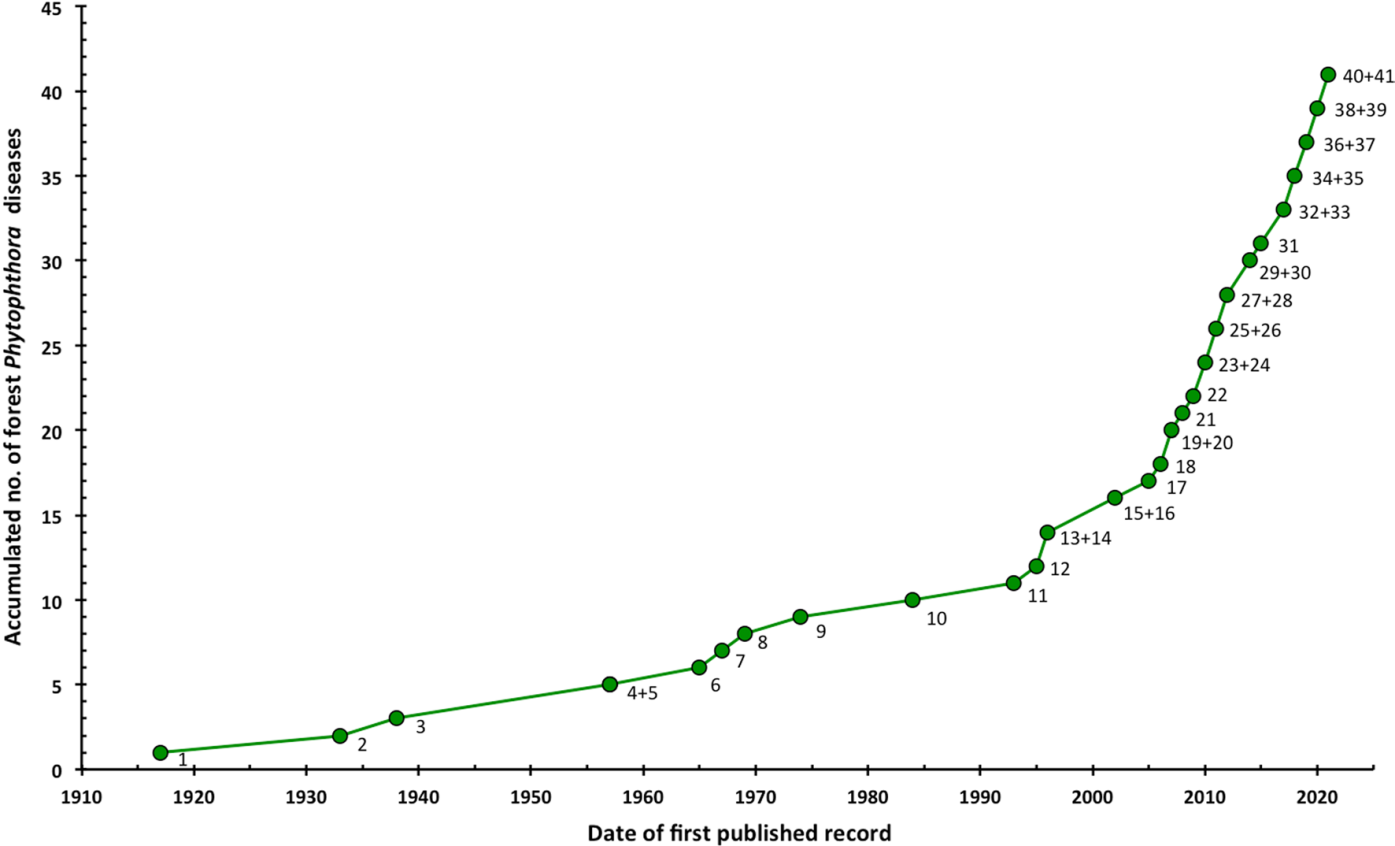
Number of important *Phytophthora* declines and diebacks of forests and natural ecosystems over time. Adapted from Jung et al. (2018a). [1 = ink disease of *Castanea sativa* in Europe (observation of first typical symptoms in 1838), 2 = ink disease of *Castanea dentata* in the USA (observation of first typical symptoms in 1824), 3 = decline of *Fagus sylvatica* in the UK, 4 = littleleaf disease of pines in the USA, 5 = decline and mortality of *Chamaecyparis lawsoniana* in the Pacific Northwest, 6 = jarrah dieback in Western Australia (WA; observation of first typical symptoms in the 1920s), 7 = ink disease of *C. crenata* and chestnut hybrids in Korea, 8 = eucalypt dieback in Victoria (observation of first typical symptoms in 1935), 9 = kauri dieback in New Zealand, 10 = Dieback of *Nothofagus* forests in Papua New Guinea, 11 = Mediterranean oak decline, 12 = *Alnus* mortality in Europe, 13 = temperate European oak decline, 14 = decline of *F. sylvatica* in mainland Europe, 15 = Sudden Oak Death in California and Oregon, 16 = littleleaf disease of *Pinus occidentalis* in the Dominican Republic, 17 = mortality of *Austrocedrus chilensis* in Argentina (observation of first typical symptoms in 1948), 18 = leaf and shoot blight of eucalypt plantations in New Zealand, 19 = oak decline in the Eastern USA, 20 = root and collar rot of eucalypt plantations in South Africa, 21 = needle cast and defoliation of *Pinus radiata* in Chile, 22 = dieback of *Eucalyptus gomphocephala* in WA, 23 = dieback of riparian *Eucalyptus rudis* in WA, 24 = Sudden Larch Death in the UK, 25 = dieback of *Araucaria excelsa* in Brazil, 26 = Ash decline in Denmark and Poland, 27 = dieback of *Nothofagus* spp. in the UK, 28 = mortality of *Juniperus communis* in the UK, 29 = red needle cast of *P. radiata* in New Zealand, 30 = leaf and twig blight of *Ilex aquifolium* in Corsica and Sardinia, 31 = dieback of Mediterranean maquis vegetation, 32 = Dieback of Fagaceae-Lauraceae monsoon forests in Northern Taiwan, 33 = Dieback of subtropical Fagaceae forests in Southern Taiwan, 34 = poplar dieback in Serbia, 35 = dieback of Valdivian rainforests in Chile, 36 = gummosis of *Acacia mearnsii* plantations in Brazil, 37 = collar rot of *P. radiata* plantations in New Zealand, 38 = dieback of laurosilva cloud forests in Northern Vietnam, 39 = black butt of *Acacia mangium* plantations in Vietnam, 40 = decline of *Cinnamomum cassia* plantations in Vietnam, 41 = cankers and dieback of Western hemlock and Douglas fir in the UK.]

These impacts can also be broadly divided into economic impacts, where Phytophthoras are causing losses or damage to cash crops in agricultural, horticultural or forestry systems; environmental impacts, where mainly introduced Phytophthoras are damaging native forest or herbaceous plant communities; and social impacts where significant damage is done to human communities with outcomes ranging from starvation, death and mass migration to loss of cultural heritage (Tables 2, 3, Additional files 1, 2: Tables S1, S2). In some cases, the impact factors are multiple. For example, the introduced *P. cinnamomi* causes damage to native forests and to important Mediterranean heath ecosystems and is also a serious problem in commercial nurseries and in horticultural and forest plantations (Brasier et al. 1993; Erwin and Ribeiro 1996; Shearer et al. 2004; Jung et al. 2016, 2018a, 2020). In Australia *P. cinnamomi* is considered a key threatening process to the Australian estate under the Environment Protection and Biodiversity Conservation Act 1999. The introduction of *P. ramorum* has had a considerable impact on the ornamental nursery trade in North America and Europe, caused heavy losses of native tanoaks (*Neolithocarpus densiflorus*) in the USA and commercial larch (*Larix kaempferi*) plantations in the UK, and collateral damage to many adjacent tree and shrub species and native ericaceous heaths (e.g. Rizzo et al. 2002; Brasier and Webber 2010; Jung et al. 2016, 2018a). The loss of oak and tanoak acorns has affected native American culture (e.g. Ortiz 2008) and food sources for wildlife. Many disease syndromes or processes involve multiple *Phytophthora* species (Tables 3, Additional file 2: Table S2). Since the 1990s, the number of previously unknown *Phytophthora* declines of forests and natural ecosystems globally has increased exponentially, from 11 to currently 41 (Fig. 2).

The number of publications and citations for selected Phytophthoras is also indicated in Table 2. Again, this should not be automatically interpreted as indicating the ‘relative importance’ of a species. While they do to an extent reflect a species’ scientific profile, such statistics can also be biased by the length of time a problem has been recognised; the economic value of particular cash crops—especially food crops; exacerbation of problems by subsequent disease management or biosecurity breaches; and the often more generous research grants available in developed countries. Furthermore, publications and citations related to environmental impacts, even major impacts such as loss of Kauri pines (*Agathis robusta*) to *P. agathidicida* in New Zealand, or loss of entire species-rich heath vegetation in southwest Australia to *P. cinnamomi* (Table 2, Additional file 3: Table S3), tend to be substantially fewer, usually as a consequence of limited research funding.

### The monophyly of *Phytophthora* and the sustained structural stability of its phylogenetic clades

The first molecular phylogenetic analysis of *Phytophthora* and other oomycetes was published by Cooke et al. (2000). It was based on ITS profiles and included 50 described *Phytophthora* species. Unlike other oomycete genera such as *Pythium* or *Halophytophthora*, *Phytophthora* was revealed as a tight monophyletic cluster of eight major clades (Clades 1–8), plus two putatively more distantly related clades (Clades 9 and 10). Unsurprisingly, the clades were also shown to transcend previous morphological groupings. Strikingly, *Peronospora sparsa* clustered within *Phytophthora* Clade 4, indicating a relatively recent evolution of *Peronospora* and other DMs from *Phytophthora*. Cooke et al. (2000) suggested *Peronospora* and *Bremia* were obligate, conidial Phytophthoras, in support of an earlier proposal by Gäumann (1952).

Since then, at least 12 other molecular phylogenetic studies of *Phytophthora* have been undertaken, ranging from increasingly complex multigene analyses (Martin and Tooley 2003; Kroon et al. 2004, 2012; Blair et al. 2008; Robideau et al. 2011; Martin et al. 2014; Rahman et al. 2015; Jung et al. 2017c; Yang et al. 2017; Bourret et al. 2018; Scanu et al. 2021; Chen et al. 2022) to a genome-wide sequence-based phylogeny (Van Poucke et al. 2021). These studies have been carried out against the background of the rapidly increasing number of described *Phytophthora* species outlined above.

Despite these fresh analyses and the addition of many new species our perception of the infrageneric structure of *Phytophthora* has changed little since Cooke et al. (2000). The overall clade structure has remained stable and generally accepted. The number of major clades, i.e. those with four or more species (therefore, excluding monospecific Clades 11, 13 and 14, currently represented by *P. lilii*; the undescribed *P.* taxon mugwort; and *P. cyperi*, which is probably a DM; Ho et al. 2004; Bourret et al. 2018) has increased from ten to eleven (Jung et al. 2017c; Chen et al. 2022). The phylogenetic positions of some species have been clarified and multiple new subclades have been added.

Above all, the major *Phytophthora* clades are still confirmed to be a relatively tight, bush-like, fundamentally monophyletic, evolutionary cluster. Indeed Clades 9 and 10, considered by Cooke et al. (2000) to be more distant, are now more closely aligned with the other major clades (Jung et al. 2017c; Yang et al. 2017; Scanu et al. 2021; Van Poucke et al. 2021; Chen et al. 2022). This is in contrast to the oomycete genus *Pythium* which, beginning with Cooke et al. (2000), has been shown to be evolutionarily divergent and polyphyletic, and in consequence was split into several monophyletic genera (de Cock et al. 2015; Uzuhashi et al. 2010).

**Fig. 3:**
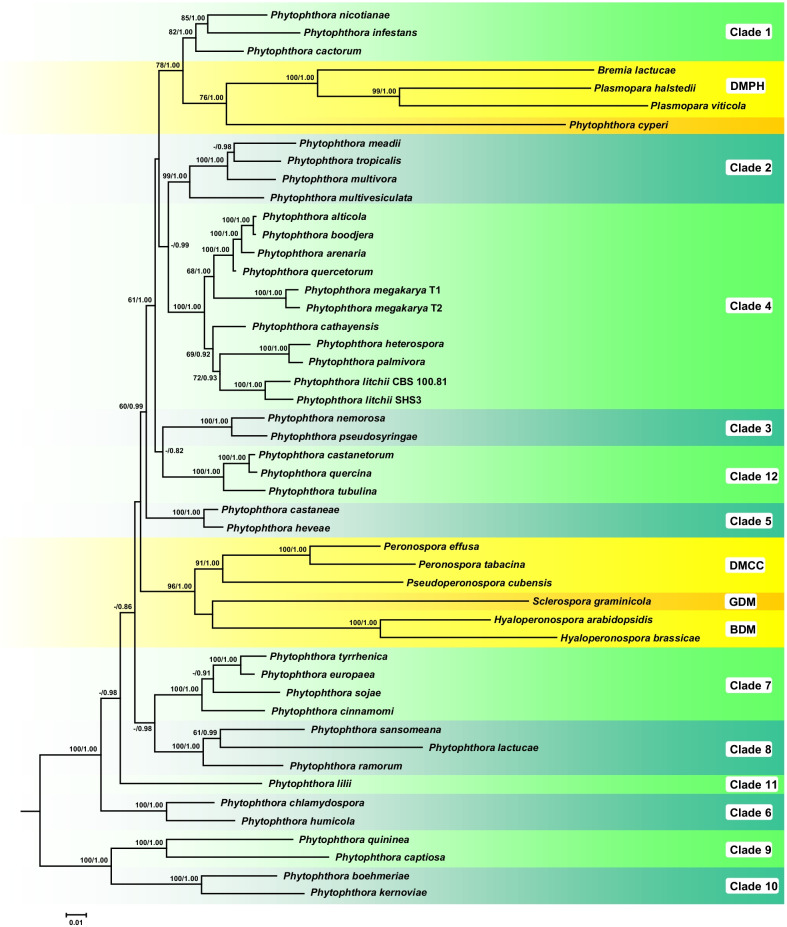
Phylogenetic tree of the *Phytophthora* clades and representative downy mildews. Redrawn from Scanu et al. (2021). A fifty percent majority rule consensus phylogram derived from maximum likelihood analysis of a concatenated four-locus (ITS, *Btub*, *cox*1, *nadh*1) dataset of representative species from phylogenetic Clades 1–12 of *Phytophthora* and the four downy mildew groups DMPH, DMCC, GDM, and BDM. Maximum likelihood bootstrap values and Bayesian posterior probabilities are indicated but not shown below 60% and 0.80, respectively. *Nothophytophthora amphigynosa* was used as outgroup taxon (not shown). Scale bar = 0.01 expected changes per site per branch

Moreover, lineages encompassing *Peronospora* and the other 19 DM genera have now been shown to have evolved from *Phytophthora* at least twice (Bourret et al. 2018; Scanu et al. 2021; Fig. 3), confirming that *Phytophthora* is ‘paraphyletic’ in relation to its DM descendants (Cooke et al. 2000). Downy mildews with pyriform haustoria (DMPHs; e.g. *Bremia*, *Plasmopara*) and the obligate biotrophic ‘*Phytophthora cyperi*’ form a monophyletic cluster in sister position to *Phytophthora* Clade 1 (Fig. 3). In contrast, those with coloured conidia (DMCCs; *Peronospora* and *Pseudoperonospora*), the graminicolous DMs (GDMs; e.g. *Sclerospora*) and the brassicolous DMs (BDMs; e.g. *Hyaloperonospora*) form a monophyletic cluster which diverged from a common ancestor with *Phytophthora* Clades 1–5, 12, and the DMPHs (Bourret et al. 2018; Scanu et al. 2021; Fig. 3). The DMs as a group therefore appear to be fundamentally polyphyletic.

### *Phytophthora* is a biologically sound and cohesive genus

**Fig. 4:**
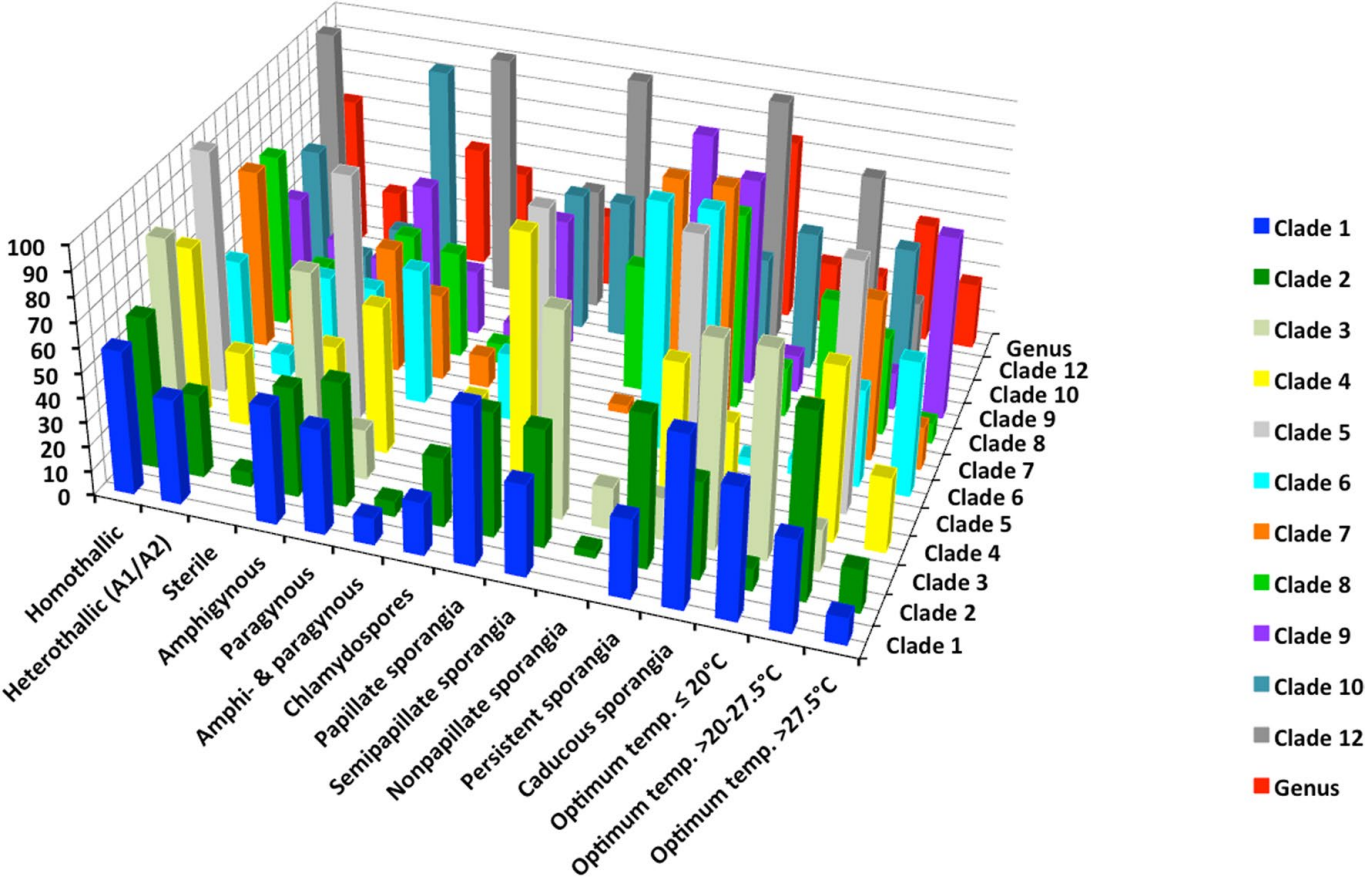
Breeding systems, main morphological characters and cardinal temperatures for growth of 196 culturable species in the 11 major *Phytophthora* clades and the genus showing percentage of species per clade or genus

**Fig. 5:**
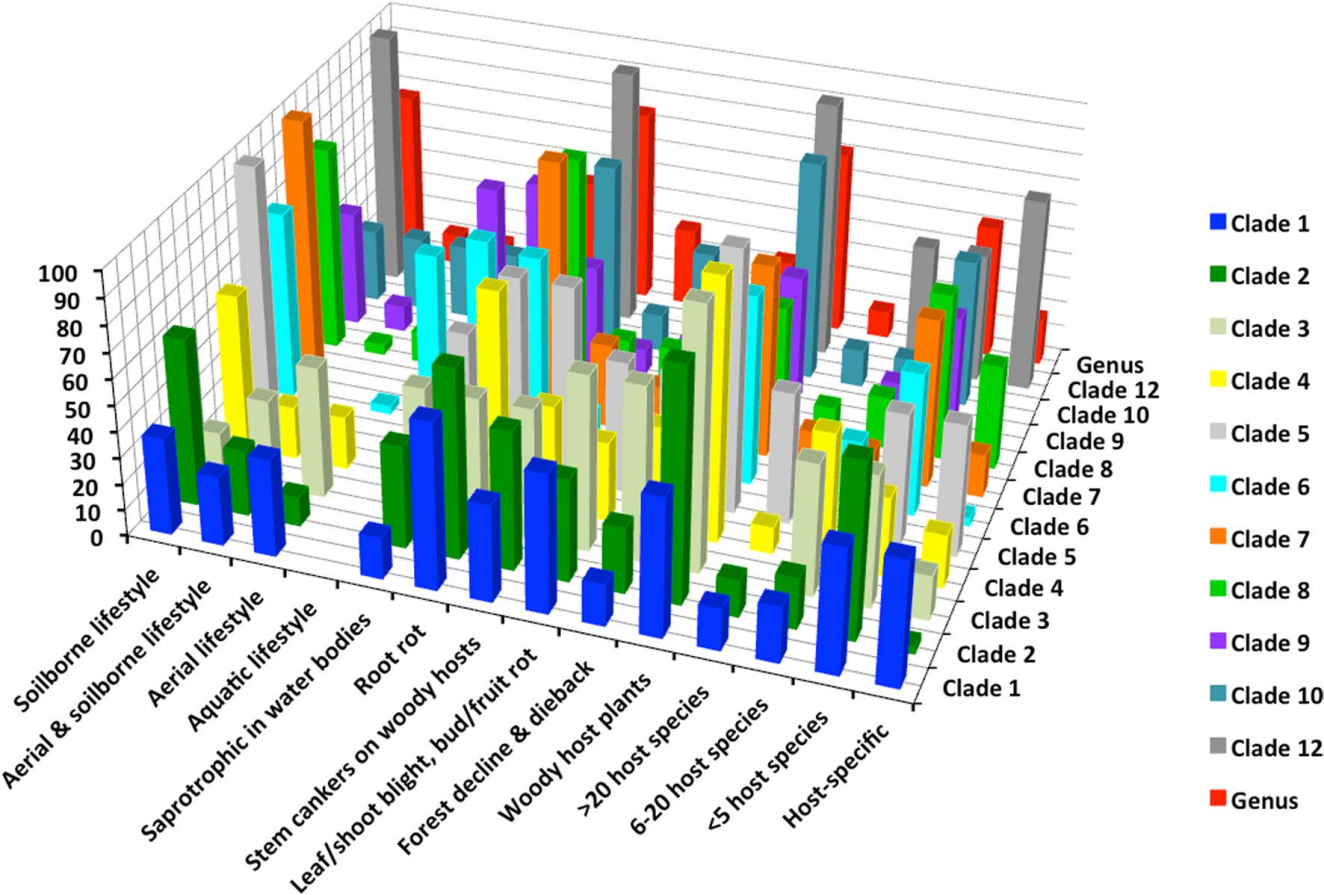
Lifestyles, diseases and host ranges of 196 culturable species in the 11 major *Phytophthora* clades and the genus showing percentage of species per clade or genus

The continued acceptance by the scientific community of *Phytophthora* as an assemblage of clades has probably also reflected a perception that this structure exhibits strong biological cohesion. Thus, the 11 major phylogenetic clades share a wide range of characters, both morphological and behavioural, that collectively characterise the genus (Figs. 4, 5; Table 4, Additional files 3, 4, 5: Tables S3, S4, S5). Also these characters often show as much variation between species within a clade as they do between clades (Table 4, Additional files 3, 4, 5: Tables S3, S4, S5).

**Table 4:**
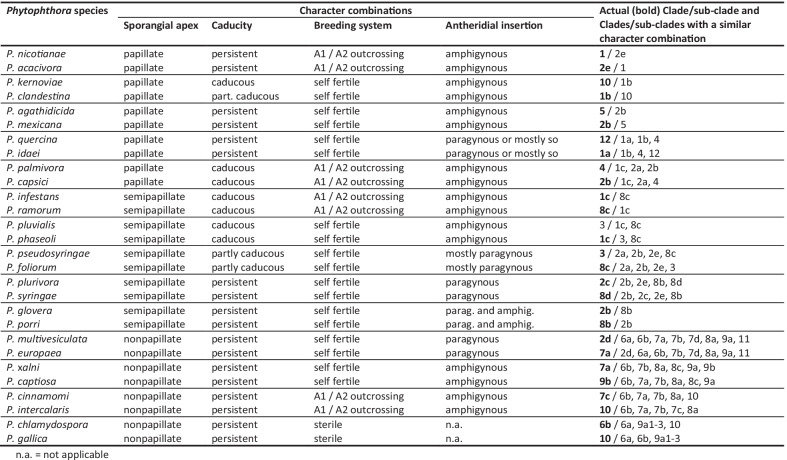
Exemplary pairs of *Phytophthora* species from phylogenetically divergent clades with similar character combinations

n.a. = not applicable

For example, caducous (deciduous) sporangia are found in nine major clades and persistent sporangia in all 11 clades (Fig. 4; Additional file 3: Table S3). Chlamydospores are produced by 59 species in ten clades; and 137 species across all eleven clades lack the ability to produce them (Fig. 4; Additional file 3: Table S3). Eight of the clades contain both self-fertile (homothallic) species and species with an A1/A2 outcrossing (heterothallic) breeding system. Both amphigynous and paragynous antheridia are found in eight clades (Fig. 4; Additional file 3: Table S3). Sterile species occur in five clades.

Conspicuous morphological similarities are shared between species in phylogenetically divergent clades (Table 4). For example, both *P. infestans* (Clade 1) and *P. ramorum* (Clade 8) have caducous, semi-papillate sporangia, and both are A1/A2 outcrossing with amphigynous antheridia. Both *P. pseudosyringae* (Clade 3) and *P. foliorum* (Clade 8) produce semipapillate, partly caducous sporangia, and both are self-fertile with mostly paragynous but some amphigynous antheridia. *Phytophthora multivesiculata* (Clade 2) and *P. europaea* (Clade 7) are both self-fertile with paragynous antheridia and both form non-papillate persistent sporangia; while *P. clandestina* (Clade 1) and *P. kernoviae* (Clade 10) are both self-fertile with amphigynous antheridia and both produce papillate caducous sporangia (Table 4).

Furthermore, phylogenetically divergent clades often share strong similarities in ‘lifestyle’. An aerial dispersal lifestyle occurs across eight and a soilborne lifestyle across all 11 major clades. Apparently very flexibly-adapted species exhibiting both an aerial and a soilborne lifestyle are found in seven clades (Fig. 5; Additional file 4: Table S4). The ability to infect and seriously damage roots, bark (phloem) and even xylem (Brown and Brasier 2007; Parke et al. 2007) tissues of woody hosts as well as herbaceous tissues is something of a *Phytophthora* speciality among the oomycetes, and largely distinguishes the genus from the obligately biotrophic DMs. It is found in 71.9% of the species and across all the clades (Fig. 5; Additional file 4: Table S4). Species with wide, medium and narrow host ranges and species exhibiting host specificity are found in all or nearly all clades (Fig. 5; Additional file 4: Table S4). In addition, currently at least 85 *Phytophthora* species (43%), representing all 11 major clades, have been shown to disperse in an aquatic environment (Fig. 5; Additional file 4: Table S4) and to live saprotrophically, free from the host. This property also distinguishes *Phytophthora* from the DMs. Forty-one species in five clades, including 25 of the 27 sterile species, have a primarily aquatic lifestyle as litter decomposers and opportunistic pathogens (Fig. 5; Additional file 4: Table S4).

A wide adaptation to climatic conditions is another feature shared across the clades: the majority contain species with either low, medium or high cardinal temperatures for growth (Fig. 4; Additional file 5: Table S5). Indeed, phylogenetically divergent Clades 1 and 8 are remarkably similar both in terms of the proportion of species adapted to low, medium or high optimum temperatures and in their maximum temperature tolerances (Additional file 5: Table S5). Furthermore, within each of the 21 different ‘lifestyle and behavioural categories’ listed in Additional files 4 and 5: Tables S4 and S5 the number of clades with taxa that exhibit the attribute is consistently high: average 9.1 across the eleven major clades; range 5–11.

Cooke et al. (2000) also reviewed the morphological and behavioural properties of their 50 *Phytophthora* taxa and proposed that Clades 1–5 comprised predominantly aerially dispersed species with papillate caducous sporangia and Clades 6–8 predominantly soil dispersed species with persistent non-papillate sporangia, consistent with an earlier proposal for two evolutionary trends in the genus (Brasier 1983). In the present analysis and that of Yang et al. (2017) this last proposal is no longer fully supported. Of the 75 species in Clades 1–5, for example, 32% are papillate caducous and aerial, another 30.7% are papillate persistent and soil inhabiting and the remaining 36.3% represent a mixture of attributes (Additional file 6: Table S6). For Clades 6–8 however the proposal does have support. Of 89 species, 80.9% are non-papillate persistent and soil inhabiting, compared to 11.2% semi-papillate persistent and soil inhabiting and 4.5% semi-papillate and partly caducous (Additional file 6: Table S6). Clades 1–5, therefore, appear more flexible in terms of their present day ‘lifestyle’ variability than Clades 6–8.

Across the clades unusual developmental features are exhibited by a small number of species (Table 5, Additional file 7: Table S7). Collectively, these are another indication of the behavioural adaptability of the genus. Sporangiophore constrictions are found in *P. pinifolia* (Clade 6) and *P. constricta* (Clade 9), presumably to facilitate aerial dispersal in otherwise non-papillate, soil and waterborne species. These appear to be an example of convergent evolution (Rea et al. 2011), as do the ultra-long sporangial pedicels produced by *P. capsici* (Clade 2) and *P*. *hibernalis* (Clade 8) (Kunimoto et al. 1976; Erwin & Ribeiro 1996). These pedicels may promote sporangial clustering (cf. Granke et al. 2009) and adherence to surfaces. Also unusual are the stromata formed by *P. cinnamomi* (Clade 7) and by *P. ramorum* and *P. lateralis* (Clade 8) (Table 5; Moralejo et al. 2006; Brasier et al. 2010; Jung et al. 2013), which may be adaptations for nutrient storage and eruption through tough leaf or periderm surfaces.

**Table 5:** Unusual morphological or developmental features among *Phytophthora* species

Species	Clade	Features^a^	Possible adaptations
*P. infestans*	1	Sporangiophore apophyses	Mechanism of indeterminate sporangiophore growth
*P. capsici*	2	Long pedicels 30- > 100 µm Umbellate sympodia	Splash dispersal, sporangial clustering and adherence to host surfaces
*P. litchii*	4	Downy white mycelium Determinate sporangiophores and synchronous sporangial formation	Resistance to dessication on suberised fruit pericarp surface Rapid synchronized sporulation on exposed fruit pericarp surface
*P. heterospora*	4	Pseudoconidia: direct germination, no papillum; formed alongside papillate zoosporic sporangia	Adaptation to both moist and drier habitats or seasonal or diurnal climate
*P. pinifolia*	6	Narrowing of sporangiophores near sporangial bases	Facultative caducity, enabling aerial infection and dispersal in a Clade of soil- and water-borne species
*P. cinnamomi*	7	Tough mycelium Stromata Lignitubers (intracellular hyphae encased in callose layers produced by the host cell)	Competitive growth through soil and litter Nutrient storage for seasonal hibernation and subsequent sporulation Long term survival
*P. lateralis*	8	Stromata and sporangiomata	Pressure eruption through needle cuticle followed by sporulation
*P. hibernalis*	8	Long pedicels 20–80 µm	Splash dispersal, sporangial clustering and adherence to host surfaces
*P. ramorum*	8	Stromata and sporangiomata	Pressure eruption through tough leaf cuticle or fruit periderm followed by sporulation
*P. constricta*	9	Sporangiophore constrictions near the sporangial bases	Facultative caducity and aerial dispersal in a Clade of soil- and waterborne species
*P. insolita*	9	Production of oospores without antheridia (presumed gametangial apomixis)	Inbreeding mechanism. Survival in periodically dry waterways without cost of less adapted recombinant offspring

^a^Associated references are shown in Additional file 7: Table S7

### Evolution and adaptability of the *Phytophthora* clades

Considering the many ecological niches and environments it has occupied, phenotypically the genus *Phytophthora* appears to have changed remarkably little. This raises the question of the evolutionary processes that have resulted in its clades being phylogenetically divergent, yet still exhibiting strong biological and behavioural conformity coupled with high adaptability. We suggest that the clades developed as a result of the migration and worldwide radiation of an ancestral Gondwanan or pre-Gondwanan *Phytophthora* population on the emerging continents, beginning around 140 Mya and that the resulting geographic isolation led to a degree of clade divergence through genetic drift, and also local adaptation to the different hosts and parts of hosts, habitats and climatic zones on the diverging continents.

We suggest the sustained biological similarity across the clades (Figs. 4, 5; Table 4, Additional files 3, 4, 5: Tables S3, S4, S5) is due to the *Phytophthora* lifestyle and genome being highly versatile in terms of (1) switching between different spore morphologies or dispersal modes (Fig. 4; Additional file 3: Table S3); (2) a range of trophic options from saprotrophy on degraded vegetation to necrotrophy and transient biotrophy on diverse tissues of pteridophytes, gymnosperms and angiosperms (Fig. 5; Additional file 4: Table S4); (3) different breeding strategies (Fig. 4; Additional file 3: Table S3), including an inbreeding system (‘homothallism’) with probably no barrier to outbreeding (cf. Whisson et al. 1994) and an outcrossing system (A1/A2 compatibility or ‘heterothallism’) that also enables selfing (Sansome 1980; Brasier 1992; Judelson 2009); (4) an ability to rapidly modify host–pathogen recognition processes via effector molecules (Kamoun et al. 1997; Kamoun 2006) and other pathogenicity factors associated with the more rapidly evolving component of the ‘two speed’ genome (Raffaele and Kamoun 2012; Zhang et al. 2019; Dale et al. 2019); (5) rapid evolution of asexual clones via mitotic recombination and transposon induced mutagenesis (e.g. Kasuga et al. 2016; Dale et al 2019); and (6) rapid evolution via interspecific hybridisation (Brasier et al. 1999, 2004; Man in’t Veldt et al. 2012; Bertier et al. 2013; Burgess 2015; Aguayo et al. 2016; Jung et al. 2017d; Van Poucke et al. 2021). These attributes may have facilitated adaptation to new hosts and new biogeographic environments without significant biological change; often resulting in convergent evolution between otherwise geographically isolated clades (Figs. 4, 5; Table 4).

In summary, we consider that *Phytophthora* is a highly flexible and highly successful biological ‘model’ that has survived well over aeons of time, leading to molecularly detectable divergence between its clades without a marked disjunction in their general morphology or behaviour. This great adaptability is probably a major factor in their being high risk pathogens when introduced into new environments.

### Emergence of the Downy Mildews

A major development among the *Phytophthora* clades has been the emergence of the DMs. On one occasion this was from a common ancestor with Clades 1–5 and Clade 12, on the other from a common ancestor with Clade 1 (Bourret et al. 2018; Fig. 3). This has resulted in the emergence of organisms with a broadly similar range of properties, apparently via convergent evolution. Probably the most definitive characteristics of the DMs are obligate biotrophy of angiosperms, usually accompanied by intracellular haustoria; and sporangiophores of determinate growth, facilitating synchronised dispersal. Major host shifts including adaptive radiation on particular plant families such as the *Poaceae* and *Brassicaceae*, combined with high levels of host specificity on largely herbaceous plant parts, are believed to have played a major role in their emergence (Göker et al. 2007; Thines and Choi 2016; Bourret et al. 2018). Also characteristic is aerial dissemination; and, in 10 of the 20 genera, directly germinating conidiosporangia (Gäumann 1964; Hall 1996; Göker et al. 2007; Thines and Choi 2016; Fletcher et al. 2019).

Given the adaptiveness and global spread of Phytophthoras it is perhaps surprising that this process has succeeded only twice. However, these developments involved abandoning definitive *Phytophthora* properties. This includes: necrotrophic ability (Thines and Choi 2016; Fletcher et al. 2018), important in *Phytophthora* parasitism of woody tissues and resulting in DMs being more benign pathogens or even endophytes; nutrient transporters linked to saprotrophic ability, enabling Phytophthoras nutrient gain in competition with other microorganisms; loss of the ability to utilise inorganic nitrogen and sulphur (Yin et al. 2017); loss of indefinite sporangiophore development, an adaptation best suited to continuously wet or aquatic conditions; and for many DMs, loss of the mechanism of zoosporogenesis (Fletcher et al. 2018) and therefore zoospore mediated infection. Indeed, detailed comparisons of *Phytophthora* and DM genomes support synteny and a common origin but also demonstrate that DMs have lost conserved domains encoding some of these properties (Fletcher et al. 2018, 2021). The narrower host and nutrient specificity, reduction in the effector repertoire and consistently reduced pathogenicity gene complements of the DMs (Fletcher et al. 2018) could render DM species more prone to extinction in a changing environment (Thines and Choi 2016). Equally, by completing their life cycles in the more sheltered and homogeneous milieu of living plant tissues DMs are probably less exposed to competition from other microorganisms; and due to their host specificity at less ‘risk’ of interspecific hybridisation, especially compared with the ecologically more flexible Phytophthoras (Brasier 2001; Van Poucke et al. 2021).

The much longer average branch lengths in the DMs, largely distinguish them from the more tightly clustered ‘bush-like’ *Phytophthora* clades, and probably reflect a relatively rapid evolution towards enhanced host specialisation and biotrophy (Bourret et al. 2018; and Fig. 3): an evolutionary jump, perhaps driven by strong directional selection associated with ensuing host–pathogen ‘arms races’. In consequence, many early stages in the evolution of the DMs are probably lost to extinction. Indeed, despite the thousand or so extant *Phytophthora* and DM taxa, examples of prominent *Phytophthora*-like characters among the DMs and vice versa are patchy and often somewhat equivocal. Thines (2009) discusses what may be ‘intermediate taxa’ or ‘bridging taxa’. But such terms are, however, subjective and should probably be treated with caution because of: (1) the unknown progenitor taxa; (2) intervening extinctions and reticulations; (3) the possibility of convergent evolution; and (4) the lack of information on the genetic control of many characters, such as haustorial form or sporangiophore development.

Amongst Phytophthoras, the recently described *P. podocarpi* (previously *P*. taxon totara) on *Podocarpus* shoots in New Zealand shares a common ancestor with the DMCCs and their relatives (Bourret et al. 2018) but otherwise has *Phytophthora* characteristics (Dobbie et al. 2022). *Phytophthora litchii* (syn. *Peronophythora litchii*) in Clade 4 resembles DMs in producing determinate sympodial sporangiophores, a ‘downy white mycelium’ on lychee (*Litchi chinensis*) fruits, and smaller gene families (Chen 1961; Ho et al. 1984; Ye et al. 2016; Sun et al. 2017). Otherwise, *P. litchii* causes necrotic lesions, is able to grow on artificial media, and displays a typical *Phytophthora*-type zoospore release and gametangial morphology. The aerial and soil inhabiting *P. heterospora* (also Clade 4), which causes bark and root necroses on various woody host plants, produces both zoospore-releasing sporangia and directly germinating pseudoconidia (Scanu et al. 2021).

The possession of such unusual characters by *P. litchii* and *P. heterospora* does not, however, confirm them as proto-DMs. The ‘DM-like’ characteristics of *P. litchii* (Tables 5, Additional file 7: Table S7) could be evidence of a common ancestor shared with the DMs, but they could also reflect convergent evolution (Ye et al. 2016) or ancestral hybridisation. The determinate sporangiophores and the ‘downy’ mycelium could be adaptations to reduce or avoid desiccation on the exposed surface of the leathery lychee pericarp. The downy mycelium might be more refractive due to a protective cell surface hydrophobin or mucin (cf. Meijer et al. 2006). The pseudoconidia of *P. heterospora*, at face value a DM-like feature, may be an adaptation allowing extra reproductive versatility in alternating moister and drier diurnal or seasonal conditions (Scanu et al. 2021). As already discussed, there are comparable unique or unusual developmental features in other *Phytophthora* species (Table 5, Additional file 7: Table S7).

#### *Phytophthora*-like characters among the DMs

Ten of the 20 DM genera, including *Pseudoperonospora*, *Plasmopara* and *Sclerophthora* produce sporangia which release zoospores (Bourret et al. 2018). *Peronospora cyperi* (syn. *Kawakamia cyperi*), was later renamed *Phytophthora cyperi*, probably because of the reportedly *Phytophthora*-like caducous sporangia and paragynous antheridia (Erwin and Ribeiro 1996)*.* Whether *P. cyperi* produces zoospores is uncertain, but it should probably be accepted under the name *K. cyperi* based on it being a biotroph (Thines et al. 2015) and its phylogenetic status (Bourret et al. 2018; and Fig. 3).

Three monotypic graminicolous DM genera, *Graminivora*, *Poakatesthia* and *Viennotia* show features not found in other DMs. In particular, the indeterminate sporangiophores and *P. infestans*-like sporangial apophyses in *V. oplismeni,* and the occurrence of intracellular hyphal growth in *Poakatesthia penniseti*, which casts doubt on whether it is an obligate biotroph (Thines 2009). These features were suggested by Beakes and Thines (2017) to be evolutionary hangovers from *Phytophthora,* but some could also reflect convergent evolution.

Perhaps the best example of a DM with *Phytophthora*-like characters is the graminicolous genus *Sclerophthora*. This is widely presumed to be biotrophic (Kenneth 1981; Erwin and Ribeiro 1996; Thines and Choi 2016) and in molecular phylogenies exhibits the characteristic long branch length, or evolutionary jump, of the DMs (Thines et al. 2008; Bourret et al. 2018). *Sclerophthora macrospora* has *Phytophthora*-like sporangiophore and sporangial morphology, and a wide but highly specialised graminicolous, and therefore non-*Phytophthora*-like, host range (Kenneth 1981; Erwin and Ribeiro 1996; Telle and Thines 2012). Tokura (1975) reported culturing *S. macrospora* on artificial media but was unable to obtain zoospores or to infect rice seedlings with these cultures. Tokura’s observations have yet to be repeated. Any attempt to do so will hopefully include diagnosis of any resulting axenic growth with molecular markers.

Among a panoply of around 800 species across 20 DM genera it is likely that some ancestral *Phytophthora*-like characteristics will have survived as long as they did not confer a marked selective disadvantage. On the evidence of Thines (2009), retention of *Phytophthora*-like characters in the DMs is associated with particular host groups. Specifically, in *Sclerophthora* and *Viennotia* in respect of the *Poaceae*, and in ‘*P. cyperi’* the *Cyperaceae*. Perhaps these associations involved host jumps, and possibly horizontal gene transfer (cf. Brasier 1995; Bourret et al. 2018; Fletcher et al. 2021), resulting in such closed host–pathogen systems that drivers towards further adaptation were less intense.

### Proposals to split *Phytophthora* into separate genera are biologically and phylogenetically inappropriate

#### Evolutionary process versus taxonomic cladism

None of the authors of the many molecular phylogenies of *Phytophthora* published since Cooke et al. (2000) have suggested there is a case either for a merging of the clades, or for their nomenclatural designation as sub-genera or sections. This apparent acceptance of the clade structure has probably also reflected a perception of their biological cohesion and of the enormous significance of the genus for scientific communication and global biosecurity (discussed later).

Nonetheless, to resolve the paraphyly of *Phytophthora,* reflected in the evidence that the DMs have evolved from the genus (Cooke et al. 2000; Jung et al. 2017a; Bourret et al. 2018; Scanu et al. 2021), and applying the terminology of cladism (not to be confused with cladistics), Runge et al. (2011) proposed either (1) placing all DMs and *Phytophthora* species in a single genus under the oldest generic name *Peronospora,* which would require renaming all *Phytophthora* species and those in 19 DM genera, resulting in a highly heterogeneous group; or (2) the description of at least six new genera within *Phytophthora* in order to conserve the DM genera. We consider the first of these suggestions intrinsically flawed as it would assimilate the ancient ancestral genus *Phytophthora* with its broad suite of morphological and behavioural characters into its highly specialised descendant, *Peronospora*, an evolutionary absurdity. Runge et al. (2011) suggested that their second alternative, splitting *Phytophthora* into around six new genera, would be most appropriate, but only on the highly questionable grounds that it would require fewer name changes. In terms of evolutionary process, their third option, reclassifying all DMs under the parental group *Phytophthora* would probably be the most logical. However, this would be in conflict with the International Code of Nomenclature for algae, fungi and plants (ICNafp) (Turland et al. 2018), which gives the first described genus *Peronospora* nomenclatural priority (though this could be overcome by conservation of *Phytophthora* over *Peronospora*); and, most importantly, it would ignore the biological realities. Voglmayr (2008) considered that none of the above alternatives would receive broad acceptance, “representing a dilemma for classification”.

Crous et al. (2021a) have also indicated that “it can be expected that *Phytophthora* will resolve into several genera in future studies”. Whether or not the paraphyly of *Phytophthora* is considered a genuine problem, that it was necessary for Runge et al. (2011) to propose two different ‘solutions’ is of concern. In our view this highlights the artificiality and subjectivity of the taxonomic process: such an approach takes little account of the often considerable biological and phylogenetic distances between *Phytophthora* and the DMs. This raises the question whether paraphyly, the emergence of one distinct life form from another without the progenitor becoming extinct, needs to be resolved by a different taxonomy at all, but should simply be accepted as a common feature of evolution in many ancient and successful genera. Around 20% of animal and 20–50% of plant species are paraphyletic in these terms (Crisp and Chandler 1996; Ross 2014). This makes it a common trait of evolution to be accepted at all taxonomic levels. Indeed, the somewhat negative use of the term ‘paraphyly’ in cladistics has been characterised as “Disparaging phylogenetic jargon for a cladogram’s representation of a progenitor in a macroevolutionary series” (Zander 2013).

Further, in terms of the definitions of Ashlock (1971) and Aubert (2015), based on the original definition of a phylon as the totality of organisms ‘related by blood and descended from a common typical ancestor’ (Haeckel 1877), *Phytophthora* is clearly monophyletic because all known *Phytophthora* species share the same common ancestor; yet *Phytophthora* is also paraphyletic because it does not contain all descendants of the common ancestor, i.e. it does not include the DMs. Collectively, however, *Phytophthora* and the DMs are holophyletic, since they contain all the descendants of their shared common ancestor. In contrast to this essentially Darwinian definition of monophyly, cladism does not discriminate between monophyly and holophyly, but focusses the definition of monophyly on the descendants, not on the ancestor as in the original Haeckelian sense (Hennig 1966; Ashlock 1971; Hörandl 2006, 2007; Aubert 2015). As a consequence, in cladism the terms monophyly and paraphyly are sometimes applied to phylogenetic trees as if they describe fixed or immutable entities. In reality they are useful generalisations that attempt to define a complex or continuum but are often unreliable due to past reticulations, significant gaps due to extinctions and, in the case of *Phytophthora*, the numerous undiscovered taxa (Brasier 2009). This renders the question of applying taxonomic weight, or names, to phylogenetic dichotomies contentious.

We consider that the key question for determining an appropriate taxonomy is not the existence of a node, but the evolutionary processes that gave rise to the divergence of the lineages and the extent of the biological changes involved (cf Brasier 2009). This approach takes into account the main geographic, genetic or biological drivers of the dichotomy. Often these can only be retrospectively inferred, rather than critically established. In terms of many analysts (e.g. Ashlock 1971; Crisp and Chandler 1996; Brummit 1998; Mayr and Bock 2002; Brummit and Sosef 2003; Hörandl 2006, 2007; Zander 2013; Aubert 2015), this is an evolutionary as opposed to a purely cladistic approach to establishing a meaningful and practical, if still approximate, taxonomy; and one in which classification is not allowed to trump evolution. Zander (2013) has pithily summarised this viewpoint as follows: *Phylogenetics imposes a classification on the results of cladistic analysis without a process-based explanation of those results. The sister-group structure is taken to be a classification itself. Evolution is not clustering, classification is. Evolution is not nesting, classification is. Phylogenetics leaps from the clustering and nesting of cladistic analysis straight to classification without explanation of the analysis in terms of serial transformations of one taxon into another, which is the nut of (Darwinian) macroevolutionary theory*.

On the above basis and taking into account our suggestions regarding the post-Gondwanan expansion of the *Phytophthora* clades, we consider *Phytophthora* to be a fundamentally monophyletic cluster that has at least twice given rise to the evolution of descendants with a distinct set of biological traits linked to obligate biotrophy, the DMs, via paraphyletic ‘jumps’. Since such jumps are common in nature, we see no current biological justification or systematic need for subjecting *Phytophthora*, a successful, ancient and biologically coherent mother genus, to segregation into separate genera. Further, proposals to do so appear mainly to be a device to address the convergent evolution of the DMs rather than a problem related to the structure of *Phytophthora *per se*.* This, despite the fact that it has also been acknowledged by Crous et al. (2021b) that, as a unit, a ‘genus’ should be defined not only by phylogeny but also by common morphological, ecological and chemical properties (= synapomorphies).

Further, because of their substantial differences in lifestyle, we consider it appropriate to accept a much broader generic concept for *Phytophthora* than for the DMs. In the more morphologically limited and behaviourally specialized DMs, genera have tended to be discriminated by conidiosporangial pigmentation and conidiophore and haustorial morphology, host specificity and, more recently, phylogenetic separation (e.g. Göker et al. 2003, 2007; Voglmayer 2008; Thines et al. 2015). For example, the BDM genus *Hyaloperonospora* is distinguished from the DMCC genus *Peronospora* largely by globose haustoria, non-pigmentation of the conidial walls and brassicaceous *versus* broad host specialisation. The other BDM genus, *Perofacia*, is distinguished from *Hyaloperonospora* largely by uniformly ellipsoidal conidia and the pseudo-dichotomous and appressed conidiophores (Constantinescu and Fatehi 2002). These generic differences are even more limited than the differences between some phylogenetically very closely related *Phytophthora* species. Thus *P. ramorum* and *P. hibernalis* in *Phytophthora* Clade 8c differ in sporangial pedicel morphology, the presence of chlamydospores, breeding system, antheridial type, oospore size, optimum temperature for growth and their main host families.

Overall, we consider that the biological and evolutionary case for retaining *Phytophthora* as a single genus is overwhelming*.* We have already shown that the 11 major *Phytophthora* clades share a characteristic diversity and plasticity across an extensive suite of morphological features, breeding systems and lifestyles. While there are trends, none of the clades are distinguished by a unique special character (synapomorphy) or combination of characters (cf. Bennett et al. 2017). In addition to monophyly, we consider the latter should be an indispensable requirement for recognition of a separate genus. Consequently, we propose for *Phytophthora*, rather than a monophyly-centred cladistic concept, a more 'Darwinian ‘ generic concept based on similarity (synapomorphies) and common descent (monophyly in the original Haeckelian sense; Mayr and Bock 2002; Hörandl 2006). This allows similarities within the older parental group (*Phytophthora*) which exists in parallel to the descendent group (DMs) while excluding similarities resulting solely from convergent evolution.

### Scientific communication and biosecurity importance of *Phytophthora* as a cohesive genus

Through being both well biologically defined and widely accepted the generic name *Phytophthora* is currently an engine of understanding and communication for a large body of scientists operating across disciplines ranging from mycology and plant pathology to biosecurity and social history. This wider ‘*Phytophthora* community’ has, over time, not only generated dedicated books (e.g. Erwin et al. 1983; Lucas et al. 1991; Erwin and Ribeiro 1996; Lamour 2013) but an enormous scientific literature base. Currently there are ca 14,000 articles in Scopus (compared with ca 4000 for all 20 DM genera combined), and that literature continues to grow rapidly. This unifying scientific communication value would be seriously damaged by an inappropriate and unnecessary attempt to break up the genus.

The impact on biosecurity needs to be seen in the context of the extensive damage caused by *Phytophthora*s to cash crops and forests (Tables 2, 3, Additional files 1, 2: Tables S1, S2); the new epidemics and pandemics resulting from introductions of exotic Phytophthoras via international trade in plants and international travel (Fig. 2); and the many new *Phytophthora* species being discovered in underexplored ecosystems with the potential to cause further pandemics, especially in host plants they have not previously encountered. Because of these threats, coherent unambiguous communication about the genus is extremely important for developing sound, evidence-based biosecurity and plant health protocols at both the international and local scale. The current understanding among regulators and scientists about what is meant by *Phytophthora* when developing plant health regulation is a valuable asset in crop and habitat protection. An unnecessarily designation of multiple new genera could seriously damage this understanding, resulting in confusion and, at worse, weakened biosecurity, adding another threat to an already fragile global environment. These problems would probably be further exacerbated by the often long time lags between taxonomic changes and names being incorporated into plant health legislation or into extension programmes.

Regarding the scope for engendering confusion, many natural and managed ecosystems are inhabited by multiple *Phytophthora* species. For example, 27 and 39 *Phytophthora* species respectively, from seven clades in each case, have recently been found in the forests and natural ecosystems of Taiwan and Vietnam (Brasier et al. 2010; Jung et al. 2017b, d, 2020); while horticultural nurseries across Europe are infested by at least 65 *Phytophthora* taxa from nine clades (Table 3, Additional file 2: Table S2; Moralejo et al. 2009; Jung et al. 2016). Moreover, the same disease syndromes are often caused by multiple Phytophthoras. At least 26 *Phytophthora* species from nine clades are associated with the current pan-European declines of oak forests (Table 3, Additional file 2: Table S2); and at least 51 species and hybrids are associated with damage to native plant communities around San Francisco (Table 3, Additional file 2: Table S2). So common are Phytophthoras on trees that commercial lateral flow devices are available to diagnose the genus (e.g. Tomlinson et al. 2010). Any designation of new genera that unnecessarily dissected the common biological properties of Phytophthoras would negatively impact communication and management in these and many similar situations and cause confusion among practitioners such as farmers, horticulturalists, forest managers and nursery owners reliant on scientific extension programmes for guidance.

The potential impact on verbal discourse deserves its own consideration. At a recent meeting of pathologists, forest health surveyors and plant health regulators in Britain addressing a previously unrecorded *Phytophthora* attacking Western Hemlock (*Tsuga heterophylla*) (Pérez-Sierra et al. 2022), discussion ranged across ten forest *Phytophthora* species from six different clades. To have referred to these by multiple generic names would have rendered the discussion unnecessarily complex and confusing to taxonomists and non-taxonomists alike. Similarly, upwards of 70 *Phytophthora* species across all 11 major clades are routinely discussed at the biennial International Forest *Phytophthora* Symposia. These meetings are an important channel of communication at a time of major *Phytophthora* threats to forests and natural ecosystems. Any unnecessary break-up of the genus would seriously undermine the value and purpose of this research community.

Numerous Government and NGO websites worldwide are dedicated to *Phytophthora* threats (e.g. Anonymous 1–4). Furthermore, terms such as ‘*Phytophthora* dieback’, ‘*Phytophthora* decline’ ‘*Phytophthora* root rot’, ‘*Phytophthora* collar rot’, ‘*Phytophthora* leaf blight’ and ‘*Phytophthora* bleeding canker’ are used widely in books, scientific papers and at meetings to discriminate the main disease syndromes common across the *Phytophthora* clades. The differences of meaning between the terms are well understood by academic plant pathologists, field surveyors and plant health regulators alike. In Britain and Australia terms such as ‘*Phytophthora* root rot’ and ‘*Phytophthora* dieback’ are even in common usage in horticultural magazines and the popular media. Broadly, these terms are an important component of the language centred around *Phytophthora* behaviour, disease management and biosecurity.

The risks to effective scientific communication inherent in over-zealous application of formal taxonomic practices in the context of molecular phylogenetics can be seen in the recent debate around *Fusarium*, another historic pathogen genus of high biosecurity importance*.* Regrettably, there now appears to be a damaging split in the international *Fusarium* community over what does, and does not, constitute the genus (Crous et al. 2021b; Geiser et al. 2021). A similar damaging controversy centred recently on the issue of whether to split the genus *Aspergillus* into multiple genera (Pitt and Taylor 2016) or retain it as a single genus (Samson et al. 2017).

## CONCLUSIONS AND RECOMMENDATIONS

Arguably *Phytophthora* remains one of, if not the best-known and most important genus of plant pathogens. Despite the rapid increase in the number of described *Phytophthora* species and improvements in molecular phylogeny the genus has remained structurally coherent and biologically well understood. In our view, no one *Phytophthora* major clade or combination of clades exhibits a sufficiently distinct set of biological characteristics to warrant a unique generic status. Paraphyletic jumps, such as the emergence of the DMs from *Phytophthora* ancestors, should be considered a normal feature of evolution in ancient and successful genera such as this. Enthusiasm to ‘dice and slice’ along the lines of cladistic nuances should not trump evolutionary or biological coherence or overlook the fact that the primary purpose of names is to facilitate communication.

We are aware that under the orthodoxies, idiosyncrasies and sometimes vague constructs of the ICNafp (cf. Hawksworth 2020) a taxonomic restructuring of *Phytophthora* could be published relatively unchallenged by any author or group regardless of their familiarity with the genus as long as they follow certain somewhat subjective rules. ‘*Phytophthora*’ is surely now bigger than all of us. In which case its status needs to be policed by a wide consensus of the scientific community, perhaps through a recommendation of a working group of the International Commission on the Taxonomy of Fungi (ICFT). We contend therefore that any proposal for a major restructuring of the circumscription of the genus should be presented to and considered by an international working group of *Phytophthora* researchers, perhaps under ICTF auspices. There are already strong precedents for the international community coming together on aspects of *Phytophthora* research. For example, an international meeting debated the case ‘for or against diploidy in *Phytophthora*’ at the University of Bari, Italy in May 1972 (Brasier 2008); and there have been large International Symposia on *Phytophthora* at the University of California, Riverside in 1982 (Erwin et al. 1983) and at Trinity College, Dublin in 1989 (Lucas et al. 1991). The issue might also be usefully addressed by a special session during the next International Mycological Congress in Maastricht in 2024.

While a case might be made for assigning sub-generic or section names to the various *Phytophthora* clades, as is the practice in some other large genera such as *Agaricus*, *Aspergillus*, *Cladonia*, *Hebeloma* and *Penicillium,* we doubt this would add much to our communication or understanding, and could be even more confusing to end users.

Considering all the above issues, and especially the lack of unequivocal evidence that defining *Phytophthora* clades as discrete genera would result in more biologically meaningful entities, we recommend that the current broad generic concept be retained. This would preserve the cultural history of the genus. It would also maintain the currently enormously effective value of the name *Phytophthora* in scientific communication, including for the many applied biologists and regulators dealing with Phytophthoras on a daily basis.

## Supplementary Information

**Additional file 1: Table S1.** Examples of the ecological, economic, social and scientific impacts of selected *Phytophthora* species.

**Additional file 2: Table S2.** Examples of the ecological, economic and social impacts of disease syndromes or processes involving multiple *Phytophthora* species.

**Additional file 3: Table S3.** Main morphological characters and breeding systems of 196 culturable *Phytophthora* species in the different clades (number/percentage of species per clade).

**Additional file 4: Table S4.** Lifestyles, diseases and host ranges of 196 culturable *Phytophthora* species in the different clades (number/percentage of species per clade).

**Additional file 5: Table S5.** Optimum and maximum temperatures for growth of 196 culturable *Phytophthora* species in the different clades (number/percentage of species per clade).

**Additional file 6: Table S6.** Sporangial characteristics of 164 species within *Phytophthora* Clades 1–5 and Clades 6–8.

**Additional file 7: Table S7.** Unusual morphological or developmental features among *Phytophthora* species.

## Data Availability

Not applicable.
