# Supplementary material for: Phytophthora: an ancient, historic, biologically and structurally cohesive and evolutionarily successful generic concept in need of preservation
Source: IMA Fungus. 2022 Jun 27;13:12. doi: 10.1186/s43008-022-00097-z (PMC9235178; doi:10.1186/s43008-022-00097-z)
Supplement: Supplementary file 1 — Additional file 1: Table S1. Examples of the ecological, economic, social and scientific impacts of selected Phytophthora species. [file 43008_2022_97_MOESM1_ESM.docx]

**Table S1**. Examples of the ecological, economic, social and scientific impacts of selected *Phytophthora* species

| ***Phytophthora* species** | **Clade** | **First described** | **Environments** | **Main diseases caused and impacts** | **Scopus indexed articles and their citations** |
| --- | --- | --- | --- | --- | --- |
| *P. agathidicida* ^a^ | 5 | 2015 | Forest, park | Dieback of Kauri, one of the world’s largest and longest-living conifer species, in New Zealand, spreading since 1974. Negative impact on both forest ecosystems and Mauri society due to the ecological and cultural significance of Kauri trees. | 29 / 171 |
| *P. austrocedi* ^b^ (syn. *P. austrocedrae*) | 8 | 2005 | Forest, natural ecosystem | Dieback and mortality of native *Austrocedrus* forests in the southern Andes. Dieback and mortality of native Juniper, UK, initially associated with restoration planting of infested nursery stock. | 26 / 135 |
| *P. cactorum* ^c^ | 1 | 1886 | Agriculture, forest, nursery | Root, collar, crown and fruit rots and stem cankers on over 200 species of trees, ornamentals, and fruit crops in 160 genera worldwide. | 586 / 6,481 |
| *P. capsici* ^d^ | 2 | 1922 | Horticulture | Phytophthora blight of *Capsicum* in the Americas and Southeast Asia, and a major limiting factor to vegetable production globally, especially cucurbits, tomatoes, and succulent beans, causing up to 100% losses in individual fields. | 1,559 / 18,150 |
| *P. cinnamomi* ^e^ | 7 | 1922 | Forest, heathland, nursery, garden | Dieback of eucalypt forests and woodlands and mass destruction of World Heritage heath flora in Western Australia since 1950s. Heavy mortality of Fagaceae in forests of southeastern US since 1940s and southern Europe since ~1990s. Damage to ornamental nursery trade in Europe since 1970s. Listed as one of the 100 worst invasive alien species; pathogenic to ~5000 trees, woody ornamentals, and herbaceous plants worldwide. | 1,331 / 12,976 |
| *P. cryptogea* ^f^ | 8 | 1919 | Horticulture, nursery, garden | Root and collar rot on a wide range of crops, fruit trees and ornamentals worldwide. Particularly important pathogen in greenhouses. | 272 / 5,261 |
| *P. fragariae* ^g^ | 7 | 1940 | Horticulture | Red core root disease of strawberry since 1920s, causing serious economic losses in strawberry plantations across humid regions of Europe and North America, with severely reduced yields and small poor-quality fruit. In Canada production losses to growers of Can$ 1,500 per ha. | 178 / 2,091 |
| *P. infestans* ^h^ | 1 | 1876 | Agriculture, horticulture | Late blight of potato and tomato, notorious for the Irish potato famine 1845-1849 resulting in mass starvation and migration. Currently still a serious threat to global food security worldwide, with US$ 6.7 million annually in yield losses and control costs. | 4,241 / 44,346 |
| *P. kernoviae* ^i^ | 10 | 2005 | Forest, heathland, horticulture, park | Aerial bleeding cankers on European beech and leaf and shoot blights of *Rhododendron,* *Magnolia* spp., and wild bilberry in the UK and Ireland. | 59 / 976 |
| *P. lateralis* ^j^ | 8 | 1942 | Forest, nursery, park, shelterbelt | Root disease causing heavy mortality of Port Orford cedar (*Chamaecyparis lawsoniana*) in its native range in Oregon and California since 1950s. Serious impact on trade in this valuable commercially harvested timber. Recently spread to ornamental *C. lawsoniana* in western Europe. Social impacts through loss of business in nursery and forestry sectors. | 62 / 1,471 |
| *P.* [*megakarya*](https://en.wikipedia.org/wiki/Phytophthora_megakarya) ^k^ | 4 | 1979 | Agroforestry | Main cause of Black pod disease of cocoa trees in central west Africa since the early 1900s, recently spread to Ghana. Loss of yield often >30% for the economically important cocoa industry, worth ca US$ 70 billion annually. | 106 / 2,301 |
| *P. nicotianae* ^l^  *(*syn. *P. parasitica)* | 1 | 1896 | Agriculture, horticulture, nursery, garden | Severe diseases of agricultural and horticultural crops worldwide, including foot rot and gummosis of citrus, black shank of tobacco and collar rot of tomato. Also on ornamentals. Broad host range, infects >255 genera in 90 plant families. | 1,235 / 17,464 |
| *P. palmivora* ^m^  (syn. *P. arecae*) | 4 | 1919 | Agroforestry, nursery, garden | Major impact on the production of tropical tree crops including black stripe disease of rubber in Southeast Asia since early 1900s. Also Black pod disease of cocoa in Southeast Asia and the Caribbean, with annual global losses to the cocoa industry of ca 450,000 t valued at > US$ 1 billion. Many ornamental hosts. | 567 / 5,892 |
| *P. plurivora* ^n^ | 2 | 2009 | Forest, nursery, park, garden | Root and collar rot and aerial stem cankers on a wide range of woody hosts in Europe and North America; involved in the decline of oak and beech across Europe. Severe impact on the ornamental nursery industry. | 66 / 644 |
| *P. quercina* ^o^ | 12 | 1999 | Forest, park | Host-specific fine root pathogen. A main driver of the chronic decline of oak forests across Europe, interacting with climatic extremes. | 53 / 896 |
| *P. ramorum* ^p^ | 8 | 2001 | Forest, nursery, garden | Over 200 plant hosts. High impact. Cause of Sudden oak death (native tanoak and other species) in the Western US since ~2000. Through loss of tanoak seed production, a significant impact on local wildlife and native American culture. Cause of Sudden larch death in the UK and Ireland since ~2010 with ~200 km^2^ plantation larch affected and millions felled Currently a threat to commercial timber production in the US (>US$ 30 billion) and the UK. Also damaging to the ornamental nursery trade in Europe and North America e.g. the rhododendron export trade in Canada (around US$ 5 million). | 627 / 8,560 |
| *P. rubi* (syn. *P. fragariae* var. *rubi*) ^q^ | 7 | 2007 | Horticulture | Extremely serious disease of raspberry plantations in Europe, North America, and elsewhere. EPPO A2 list, recommended for phytosanitary treatments. | 57 / 475 |
| *P. sojae* (syn. *P. megasperma* var. *sojae*) ^r^ | 7 | 1958 | Horticulture | Devastating root and stem rot of soybean in the US, with an annual cost worldwide of US$ 1-2 billion. | 810 / 13,592 |
| *P. syringae* ^s^ | 8 | 1909 | Horticulture, nursery, garden | Root and collar rot, stem cankers, leaf and shoot blights and fruit rot on a medium-wide range of host plants including fruit trees and lilac. | 71 / 2,207 |
| *P.* ×*alni* ^t^ | 7 | 2004 | Riparian forest, nursery | Extensive mortality of riparian alder across Europe since 1990s, driven by planting of infested nursery stock. Impacts ecosystem functions and services and riverbank stability. EPPO alert list 1996 to 2001. | 72 / 772 |
| *P.* ×*cambivora* ^u^ | 7 | 1927 | Forest, horticulture, nursery, garden | Root and collar infections (Ink disease) of sweet chestnut and beech in Europe. Root rot of various fruit trees in Europe and the US since 1900s. Significant impact on ornamental nurseries. | 142 / 2,259 |

**References**

^a^ Scott PM, Williams N (2014) Phytophthora diseases in New Zealand forests. New Zealand Journal of Forestry 59: 14–21.

Weir BS, Paderes EP, Anand N, Uchida JY, Pennycook SR, Bellgard SE, Beever RE (2015) A taxonomic revision of *Phytophthora* Clade 5, including two new species, *Phytophthora agathidicida* and *P. cocois*. Phytotaxa 205:21–38.

^b^ Green S, Elliot M, Armstrong A, Hendry SJ (2015) *Phytophthora austrocedrae* emerges as a serious threat to juniper *Juniperus communis* in Britain. Plant Pathol 64:456–466.

Greslebin AG, Hansen EM, Sutton W. 2007. *Phytophthora austrocedrae* sp. nov., a new species associated with *Austrocedrus chilensis* mortality in Patagonia Argentina. Mycol Res 111:308–316.

^c^ Erwin DC, Ribeiro OK (1996) Phytophthora diseases worldwide. American Phytopathological Society (APS Press), St. Paul, Minnesota:562 pp.

Jung T, Orlikowski L, Henricot B, Abad-Campos P, Aday AG, Aguín Casal O, Bakonyi J, Cacciola SO, Cech T, Chavarriaga D, Corcobado T, Cravador A, Decourcelle T, Denton G, Diamandis S, Doğmuş-Lehtijärvi HT, Franceschini A, Ginetti B, Green S, Glavendekić M, Hantula J, Hartmann G, Herrero M, Ivic D, Horta Jung M, Lilja A, Keca N, Kramarets V, Lyubenova A, Machado H, Magnano di San Lio G, Mansilla Vázquez PJ, Marçais B, Matsiakh I, Milenkovic I, Moricca S, Nagy ZÁ, Nechwatal J, Olsson C, Oszako T, Pane A, Paplomatas EJ, Pintos Varela C, Prospero S, Rial Martínez C, Rigling D, Robin C, Rytkönen A, Sánchez ME, Sanz Ros AV, Scanu B, Schlenzig A, Schumacher J, Slavov S, Solla A, Sousa E, Stenlid J, Talgø V, Tomic Z, Tsopelas P, Vannini A, Vettraino AM, Wenneker M, Woodward S, Peréz-Sierra A (2016) Widespread *Phytophthora* infestations in European nurseries put forest, semi-natural and horticultural ecosystems at high risk of Phytophthora diseases. Forest Pathol 46:134–163.

Jung T, Pérez-Sierra A, Durán A, Jung MH, Balci Y, Scanu B (2018) Canker and decline diseases caused by soil- and airborne *Phytophthora* species in forests and woodlands. Persoonia 40:182–220.

^d^ Erwin DC, Ribeiro OK (1996) Phytophthora diseases worldwide. American Phytopathological Society (APS Press), St. Paul, Minnesota:562 pp.

Granke L, Quesada-Ocampo L, Hausbeck M (2013*) Phytophthora capsici* in the Eastern USA. In: Lamour K (ed), Phytophthora: A global perspective: CABI, Wallingford, UK:96–103.

^e^ Brasier CM (1992) Oak tree mortality in Iberia. Nature 360: 539.

Brasier C.M, Robredo F, & Ferraz JFP (1993). Evidence for *Phytophthora cinnamomi* involvement in Iberian oak decline. Plant Pathol 42: 140-145.

Brasier CM (1996) *Phytophthora cinnamomi* associated oak decline: Environmental constraints including climate change. Ann Sciences Forest 53: 347–358.

Crandall BS, Gravatt GF, Ryan MM (1945) root disease of *Castanea* species and some coniferous and broadleaf nursery stocks caused by *Phytophthora cinnamomoi.* Phytopathol 35; 162–180.

Erwin DC, Ribeiro OK (1996) Phytophthora diseases worldwide. American Phytopathological Society (APS Press), St. Paul, Minnesota:562 pp.

Grente J, Solignat G (1952) La maladie de l’encre du Chataigner et son evolution. A R Acad Agric Fr 38: 126–129.

Hardham AR, Blackman LM (2018) *Phytophthora cinnamomi*. Mol Plant Pathol 19: 260–285.

Jung T, Orlikowski L, Henricot B, Abad-Campos P, Aday AG, Aguín Casal O, Bakonyi J, Cacciola SO, Cech T, Chavarriaga D, Corcobado T, Cravador A, Decourcelle T, Denton G, Diamandis S, Doğmuş-Lehtijärvi HT, Franceschini A, Ginetti B, Green S, Glavendekić M, Hantula J, Hartmann G, Herrero M, Ivic D, Horta Jung M, Lilja A, Keca N, Kramarets V, Lyubenova A, Machado H, Magnano di San Lio G, Mansilla Vázquez PJ, Marçais B, Matsiakh I, Milenkovic I, Moricca S, Nagy ZÁ, Nechwatal J, Olsson C, Oszako T, Pane A, Paplomatas EJ, Pintos Varela C, Prospero S, Rial Martínez C, Rigling D, Robin C, Rytkönen A, Sánchez ME, Sanz Ros AV, Scanu B, Schlenzig A, Schumacher J, Slavov S, Solla A, Sousa E, Stenlid J, Talgø V, Tomic Z, Tsopelas P, Vannini A, Vettraino AM, Wenneker M, Woodward S, Peréz-Sierra A (2016) Widespread *Phytophthora* infestations in European nurseries put forest, semi-natural and horticultural ecosystems at high risk of Phytophthora diseases. Forest Pathol 46:134–163.

Jung T, Pérez-Sierra A, Durán A, Jung MH, Balci Y, Scanu B (2018) Canker and decline diseases caused by soil- and airborne *Phytophthora* species in forests and woodlands. Persoonia 40:182–220.

Shearer BL, Crane CE, Cochrane A (2004) Quantification of the susceptibility of the native flora of the South-West Botanical Province, Western Australia, to *Phytophthora cinnamomi*. Aust J Bot 52:435–443.

Shearer BL, Tippett JT (1989) Jarrah dieback: The dynamics and manage- ment of *Phytophthora cinnamomi* in the jarrah (*Eucalyptus marginata*) forests of south-western Australia. Perth, Department of Conservation and Land Management.

Urquiljo Landaluze P (1947) Revision taxonomica de los hongos productores la enfermidad del Castano llamada ‘la tinta’. Bol. Pat. Veg. Ent. Agric. Madrid 16: 253-270.

 Weste G, Marks GC (1987) The biology of *Phytophthora cinnamomi* in Australasian forests. Ann Rev Phytopathol 25: 207–229.

^f^ Erwin DC, Ribeiro OK (1996) Phytophthora diseases worldwide. American Phytopathological Society (APS Press), St. Paul, Minnesota:562 pp.

^g^ Erwin DC, Ribeiro OK (1996) Phytophthora diseases worldwide. American Phytopathological Society (APS Press), St. Paul, Minnesota:562 pp.

Maas JL (1998) Compendium of strawberry diseases, Second Edition. American Phytopathological Society (APS Press), St. Paul, Minnesota:159 pp.

^h^ Erwin DC, Ribeiro OK (1996) Phytophthora diseases worldwide. American Phytopathological Society (APS Press), St. Paul, Minnesota:562 pp.

Cooke DEL, Andersson B (2013) *Phytophthora infestans* and Potato Late Blight in Europe. In: Lamour K (ed), Phytophthora: A global perspective: CABI, Wallingford, UK:59–67.

Halterman D, Gevens AJ (2013) *Phytophthora infestans* in the USA. In: Lamour K (ed), Phytophthora: A global perspective: CABI, Wallingford, UK:68–78.

Haverkort AJ, Boonekamp PM, Hutten R, Jacobsen E, Lotz LAP, Kessel GJT, Visser RGF, van der Vossen EAG (2008). Societal costs of late blight in potato and prospects of durable resistance through cisgenic modification. Potato Research 51:47–57.

^i^ Brasier CM, Beales PA, Kirk SA, Denman S, Rose J (2005) *Phytophthora kernoviae* *sp. nov.* an invasive pathogen causing bleeding stem lesions on forest trees and foliar necrosis of ornamentals in Britain. Mycol Res 109:853–859.

^j^ Green S, Brasier CM, Schlenzig A, McCracken A, MacAskill GA, Wilson M, Webber JF (2013) The destructive invasive pathogen *Phytophthora lateralis* found on *Chamaecyparis lawsoniana* across the UK. Forest Pathol 43:19–28.

Hansen EM, Goheen DJ, Jules ES, Ullian B (2000) Managing Port-Orford-cedar and the introduced pathogen *Phytophthora lateralis*. Plant Dis 84:4–14.

Jules ES, Kauffman MJ, Ritts WD, Carroll AL (2002). Spread of an invasive pathogen over a variable landscape: a nonnative root rot on Port Orford cedar. Ecology 83:3167–3181.

Jung T, Pérez-Sierra A, Durán A, Jung MH, Balci Y, Scanu B (2018) Canker and decline diseases caused by soil- and airborne *Phytophthora* species in forests and woodlands. Persoonia 40:182–220.

Robin C, Piou D, Feau NF, Douzon G, Schenck N, Hansen EM (2011) Root and aerial infections of *Chamaecyparis lawsoniana* by *Phytophthora lateralis*: a new threat for European countries. Forest Pathol 41:417–424.

^k^ Anonymous (1981) Epidemiology of *Phytophthora* on cocoa in Nigeria - Final report of the International cocoa black pod research project. Commonw. Mycol. Inst. Phytopathol. Pap. No. 25:188 pp.

Erwin DC, Ribeiro OK (1996) Phytophthora diseases worldwide. American Phytopathological Society (APS Press), St. Paul, Minnesota:562 pp.

Ploetz, R.C. (2007) Cacao diseases: important threats to chocolate production worldwide. Phytopathology 97: 1634–1639.

^l^ Erwin DC, Ribeiro OK (1996) Phytophthora diseases worldwide. American Phytopathological Society (APS Press), St. Paul, Minnesota:562 pp.

Ludovici VA, Zhang W, Blackman LM, Hardham AR (2013) *Phytophthora nicotianae*. In: Lamour K (ed), Phytophthora: A global perspective: CABI, Wallingford, UK:113–123.

Panabières F, Ali GS, Allagui MB, Dalio RJD, Gudmestad MC, Kuhn M-L, Guha Roy S, Schena L, Zampounis A (2016). *Phytophthora nicotianae* diseases worldwide: new knowledge of a long-recognised pathogen. Phytopathol Mediterr 55:20−40.

^m^ Anonymous (1981) Epidemiology of *Phytophthora* on cocoa in Nigeria - Final report of the International cocoa black pod research project. Commonw. Mycol. Inst. Phytopathol. Pap. No. 25:188 pp.

Erwin DC, Ribeiro OK (1996) Phytophthora diseases worldwide. American Phytopathological Society (APS Press), St. Paul, Minnesota:562 pp.

Drenth A, Guest DI (eds.) (2004) Diversity and management of *Phytophthora* in Southeast Asia. Australian Centre for International Agricultural Research, Canberra, Australia.

Drenth A, Guest D (2013) *Phytophthora palmivora* in Tropical Tree Crops. In: Lamour K (ed), Phytophthora: A global perspective: CABI, Wallingford, UK:187–196.

^n^ Jung T, Burgess TI (2009) Re-evaluation of *Phytophthora citricola* isolates from multiple woody hosts in Europe and North America reveals a new species, *Phytophthora plurivora* *sp. nov*. Persoonia 22: 95–110.

Jung T, Orlikowski L, Henricot B, Abad-Campos P, Aday AG, Aguín Casal O, Bakonyi J, Cacciola SO, Cech T, Chavarriaga D, Corcobado T, Cravador A, Decourcelle T, Denton G, Diamandis S, Doğmuş-Lehtijärvi HT, Franceschini A, Ginetti B, Green S, Glavendekić M, Hantula J, Hartmann G, Herrero M, Ivic D, Horta Jung M, Lilja A, Keca N, Kramarets V, Lyubenova A, Machado H, Magnano di San Lio G, Mansilla Vázquez PJ, Marçais B, Matsiakh I, Milenkovic I, Moricca S, Nagy ZÁ, Nechwatal J, Olsson C, Oszako T, Pane A, Paplomatas EJ, Pintos Varela C, Prospero S, Rial Martínez C, Rigling D, Robin C, Rytkönen A, Sánchez ME, Sanz Ros AV, Scanu B, Schlenzig A, Schumacher J, Slavov S, Solla A, Sousa E, Stenlid J, Talgø V, Tomic Z, Tsopelas P, Vannini A, Vettraino AM, Wenneker M, Woodward S, Peréz-Sierra A (2016) Widespread *Phytophthora* infestations in European nurseries put forest, semi-natural and horticultural ecosystems at high risk of Phytophthora diseases. Forest Pathol 46:134–163.

Jung T, Pérez-Sierra A, Durán A, Jung MH, Balci Y, Scanu B (2018) Canker and decline diseases caused by soil- and airborne *Phytophthora* species in forests and woodlands. Persoonia 40:182–220.

^o^ Jönsson U, Jung T, Sonesson K, Rosengren U (2005) Relationships between *Quercus robur* health, occurrence of *Phytophthora* species and site conditions in southern Sweden. Plant Pathol 54:502–511.

Jung T, Cooke DEL, Blaschke H, Duncan JM, Oßwald W (1999) *Phytophthora quercina* *sp. nov*., causing root rot of European oaks. Mycol Res 103:785–798.

Jung T, Blaschke H, Oßwald W (2000) Involvement of soilborne *Phytophthora* species in Central European oak decline and the effect of site factors on the disease. Plant Pathol 49:706-718.

Jung T, Orlikowski L, Henricot B, Abad-Campos P, Aday AG, Aguín Casal O, Bakonyi J, Cacciola SO, Cech T, Chavarriaga D, Corcobado T, Cravador A, Decourcelle T, Denton G, Diamandis S, Doğmuş-Lehtijärvi HT, Franceschini A, Ginetti B, Green S, Glavendekić M, Hantula J, Hartmann G, Herrero M, Ivic D, Horta Jung M, Lilja A, Keca N, Kramarets V, Lyubenova A, Machado H, Magnano di San Lio G, Mansilla Vázquez PJ, Marçais B, Matsiakh I, Milenkovic I, Moricca S, Nagy ZÁ, Nechwatal J, Olsson C, Oszako T, Pane A, Paplomatas EJ, Pintos Varela C, Prospero S, Rial Martínez C, Rigling D, Robin C, Rytkönen A, Sánchez ME, Sanz Ros AV, Scanu B, Schlenzig A, Schumacher J, Slavov S, Solla A, Sousa E, Stenlid J, Talgø V, Tomic Z, Tsopelas P, Vannini A, Vettraino AM, Wenneker M, Woodward S, Peréz-Sierra A (2016) Widespread *Phytophthora* infestations in European nurseries put forest, semi-natural and horticultural ecosystems at high risk of Phytophthora diseases. Forest Pathol 46:134–163.

Jung T, Pérez-Sierra A, Durán A, Jung MH, Balci Y, Scanu B (2018) Canker and decline diseases caused by soil- and airborne *Phytophthora* species in forests and woodlands. Persoonia 40:182–220.

Pérez-Sierra A, López-García C, León M, Garcia-Jimenez, J, Abad-Campos P, Jung T (2013) Previously unrecorded low temperature *Phytophthora* species associated with *Quercus* decline in a Mediterranean forest in Eastern Spain. Forest Pathol 43:331–339.

Seddaiu S, Brandano A, Ruiu PA, Sechi C, Scanu B (2020). An overview of *Phytophthora* species inhabiting declining *Quercus suber* stands in Sardinia (Italy). Forests 11:971.

Vettraino AM, Barzanti GP, Bianco MC, Ragazzi A, Capretti P, Paoletti E, Luisi N, Anselmi N, Vannini A (2002) Occurrence of *Phytophthora* species in oak stands in Italy and their association with declining oak trees. Forest Pathol 32:19–28.

^p^ Brasier CM, Webber J (2010) Sudden larch death. Nature 466:824–825.

Grünwald NJ, Garbelotto M, Goss EM, Heungens K, Prospero S (2012) Emergence of the Sudden Oak Death pathogen *Phytophthora ramorum*. Trends Microbiol 20:131–138.

Grünwald NJ, Goss EM, Press CM. 2008. *Phytophthora ramorum*: a pathogen with a remarkably wide host range causing Sudden Oak Death on oaks and ramorum blight on woody ornamentals. Mol Plant Pathol 9:729–740.

Jung T, Pérez-Sierra A, Durán A, Jung MH, Balci Y, Scanu B (2018) Canker and decline diseases caused by soil- and airborne *Phytophthora* species in forests and woodlands. Persoonia 40:182–220.

Rizzo DM, Garbelotto M, Davidson JM, Slaughter GW, Koike ST (2002) *Phytophthora ramorum* as the cause of extensive mortality of *Quercus* spp. and *Lithocarpus densiflorus* in California. Plant Dis 86:205–214.

Rizzo DM, Garbelotto M, Hansen EM (2005) *Phytophthora ramorum*: integrative research and management of an emerging pathogen in California and Oregon forests. Ann Rev Phytopathol 43:309–335.

Webber JF, Brasier CM (2018). Ramorum disease of larch. In: Hansen EM, Lewis KJ, Chastagner GA, eds. Compendium of Conifer Diseases. 2nd edn. St Paul, MN, USA: APS Press, pp. 13–14.

^q^ Erwin DC, Ribeiro OK (1996) Phytophthora diseases worldwide. American Phytopathological Society (APS Press), St. Paul, Minnesota:562 pp.

Wilcox WF, Scott PH, Hamm PB, Kennedy DM, Duncan JM, Brasier CM, Hansen EM (1993). Identity of a *Phytophthora* species attacking raspberry in Europe and North America. Mycol Res 97:817–831.

^r^ Dorrance AE (2013) *Phytophthora sojae* on soybean. In: Lamour K (ed), Phytophthora: A global perspective: CABI, Wallingford, UK:79–86.

Erwin DC, Ribeiro OK (1996) Phytophthora diseases worldwide. American Phytopathological Society (APS Press), St. Paul, Minnesota:562 pp.

Tyler BM (2007) *Phytophthora sojae*: root rot pathogen of soybean and model oomycete. Mol Plant Pathol 8(1):1–8.

^s^ Erwin DC, Ribeiro OK (1996) Phytophthora diseases worldwide. American Phytopathological Society (APS Press), St. Paul, Minnesota:562 pp.

Harris DC (1991) The Phytophthora disease of apple. J Hortic Sci 66:513–544.

Jung T, Orlikowski L, Henricot B, Abad-Campos P, Aday AG, Aguín Casal O, Bakonyi J, Cacciola SO, Cech T, Chavarriaga D, Corcobado T, Cravador A, Decourcelle T, Denton G, Diamandis S, Doğmuş-Lehtijärvi HT, Franceschini A, Ginetti B, Green S, Glavendekić M, Hantula J, Hartmann G, Herrero M, Ivic D, Horta Jung M, Lilja A, Keca N, Kramarets V, Lyubenova A, Machado H, Magnano di San Lio G, Mansilla Vázquez PJ, Marçais B, Matsiakh I, Milenkovic I, Moricca S, Nagy ZÁ, Nechwatal J, Olsson C, Oszako T, Pane A, Paplomatas EJ, Pintos Varela C, Prospero S, Rial Martínez C, Rigling D, Robin C, Rytkönen A, Sánchez ME, Sanz Ros AV, Scanu B, Schlenzig A, Schumacher J, Slavov S, Solla A, Sousa E, Stenlid J, Talgø V, Tomic Z, Tsopelas P, Vannini A, Vettraino AM, Wenneker M, Woodward S, Peréz-Sierra A (2016) Widespread *Phytophthora* infestations in European nurseries put forest, semi-natural and horticultural ecosystems at high risk of Phytophthora diseases. Forest Pathol 46:134–163.

^t^ Brasier CM, Kirk SA, Delcan J, Cooke DEL, Jung T, Man in’t Veld WA (2004) *Phytophthora alni* sp. nov. and its variants: designation of emerging heteroploid hybrid pathogens spreading on *Alnus* trees. Mycol Res 108:1172–1184.

Gibbs JN, Lipscombe MA, Peace AJ (1999) The impact of Phytophthora disease on riparian populations of common alder (*Alnus glutinosa*) in southern Britain. Eur J Forest Pathol 29:39–50.

Gibbs JN, Van Dijk C, Webber JF (eds). 2003. Phytophthora disease of alder in Europe. Forestry Commission Bulletin 126, Edinburgh, UK.

Jung T, Blaschke M (2004) Phytophthora root and collar rot of alders in Bavaria: distribution, modes of spread and possible management strategies. Plant Pathol 53:197–208.

Jung T, Orlikowski L, Henricot B, Abad-Campos P, Aday AG, Aguín Casal O, Bakonyi J, Cacciola SO, Cech T, Chavarriaga D, Corcobado T, Cravador A, Decourcelle T, Denton G, Diamandis S, Doğmuş-Lehtijärvi HT, Franceschini A, Ginetti B, Green S, Glavendekić M, Hantula J, Hartmann G, Herrero M, Ivic D, Horta Jung M, Lilja A, Keca N, Kramarets V, Lyubenova A, Machado H, Magnano di San Lio G, Mansilla Vázquez PJ, Marçais B, Matsiakh I, Milenkovic I, Moricca S, Nagy ZÁ, Nechwatal J, Olsson C, Oszako T, Pane A, Paplomatas EJ, Pintos Varela C, Prospero S, Rial Martínez C, Rigling D, Robin C, Rytkönen A, Sánchez ME, Sanz Ros AV, Scanu B, Schlenzig A, Schumacher J, Slavov S, Solla A, Sousa E, Stenlid J, Talgø V, Tomic Z, Tsopelas P, Vannini A, Vettraino AM, Wenneker M, Woodward S, Peréz-Sierra A (2016) Widespread *Phytophthora* infestations in European nurseries put forest, semi-natural and horticultural ecosystems at high risk of Phytophthora diseases. Forest Pathol 46:134–163.

Jung T, Pérez-Sierra A, Durán A, Jung MH, Balci Y, Scanu B (2018) Canker and decline diseases caused by soil- and airborne *Phytophthora* species in forests and woodlands. Persoonia 40:182–220.

Streito JC, Legrand P, Tabary F, de Villartay J (2002) Phytophthora disease of alder (*Alnus glutinosa*) in France: investigations between 1995 and 1999. Forest Pathol 32:179–191.

^u^ Day PR (1939) Root rot of sweet chestnut and beech caused by species of *Phytophthora*. I. Cause and symptoms of disease: its relation to site conditions. Forestry 12: 101-116.

Erwin DC, Ribeiro OK (1996) Phytophthora diseases worldwide. American Phytopathological Society (APS Press), St. Paul, Minnesota: 562 pp.

Jung T (2009) Beech decline in Central Europe driven by the interaction between *Phytophthora* infections and climatic extremes. Forest Pathol 39:73–94.

Jung T, Orlikowski L, Henricot B, Abad-Campos P, Aday AG, Aguín Casal O, Bakonyi J, Cacciola SO, Cech T, Chavarriaga D, Corcobado T, Cravador A, Decourcelle T, Denton G, Diamandis S, Doğmuş-Lehtijärvi HT, Franceschini A, Ginetti B, Green S, Glavendekić M, Hantula J, Hartmann G, Herrero M, Ivic D, Horta Jung M, Lilja A, Keca N, Kramarets V, Lyubenova A, Machado H, Magnano di San Lio G, Mansilla Vázquez PJ, Marçais B, Matsiakh I, Milenkovic I, Moricca S, Nagy ZÁ, Nechwatal J, Olsson C, Oszako T, Pane A, Paplomatas EJ, Pintos Varela C, Prospero S, Rial Martínez C, Rigling D, Robin C, Rytkönen A, Sánchez ME, Sanz Ros AV, Scanu B, Schlenzig A, Schumacher J, Slavov S, Solla A, Sousa E, Stenlid J, Talgø V, Tomic Z, Tsopelas P, Vannini A, Vettraino AM, Wenneker M, Woodward S, Peréz-Sierra A (2016) Widespread *Phytophthora* infestations in European nurseries put forest, semi-natural and horticultural ecosystems at high risk of Phytophthora diseases. Forest Pathol 46:134–163.

Jung T, Pérez-Sierra A, Durán A, Jung MH, Balci Y, Scanu B (2018) Canker and decline diseases caused by soil- and airborne *Phytophthora* species in forests and woodlands. Persoonia 40:182–220.

Mircetich SM (1982) Phytophthora root and crown rot of apricot trees. Acta Hortic 21:272–276.

Saavedra A, Hansen EM, Goheen DJ (2007) *Phytophthora cambivora* in Oregon and its pathogenicity to Chrysolepis chrysophylla. Forest Pathol 37:409–419.

Stępniewska H, Dłuszyński J (2010) Incidence of *Phytophthora* *cambivora* in bleeding lesions on beech stems in selected forest stands in south-eastern Poland. Phytopathologia 56:39–51.

Telfer KH, Brurberg MB, Herrero ML, Stensvand A, Talgø V (2015) *Phytophthora* *cambivora* found on beech in Norway. Forest Pathol 45:415–425.

Vettraino AM, Morel O, Perlerou C, Robin C, Diamandis S, Vannini A (2005) Occurrence and distribution of *Phytophthora* species associated with ink disease of chestnut in Europe. Eur J Plant Pathol 111:169–180.
