# Supplementary material for: Phytophthora: an ancient, historic, biologically and structurally cohesive and evolutionarily successful generic concept in need of preservation
Source: IMA Fungus. 2022 Jun 27;13:12. doi: 10.1186/s43008-022-00097-z (PMC9235178; doi:10.1186/s43008-022-00097-z)
Supplement: Supplementary file 2 — Additional file 2: Table S2. Examples of the ecological, economic and social impacts of disease syndromes or processes involving multiple Phytophthora species. [file 43008_2022_97_MOESM2_ESM.docx]

**Table S2.** Examples of the ecological, economic and social impacts of disease syndromes or processes involving multiple *Phytophthora* species

| **Syndrome or process and location** | **Environments** | **No. of *Phytophthora* taxa and hybrids involved** | **Clades** | **Impacts** |
| --- | --- | --- | --- | --- |
| Cocoa black pod disease:  West Africa, Caribbean, South America, Southeast Asia | Plantation | 5 ^a^ | 2, 4, 5 | Cocoa pod lesions. Heavy crop losses (cf. *P. megakarya*, Table 2). Impact on small scale local farming communities and on global chocolate industry. |
| Oak decline: across Europe | Forest, park | 26 ^b^ | 1, 2, 3, 5, 6, 7, 8, 10, 12 | Root lesions and sometimes also collar lesion leading to forest declines driven in part by introduced pathogens and interaction with climate change. Impact on forestry and recreation. |
| Beech decline: across Europe | Forest, park | 16 ^c^ | 1, 2, 3, 5, 6, 7, 10, 12 | Root lesions, collar lesions, stem lesions leading to forest declines driven in part by introduced pathogens and interaction with climate change. Impact on forestry and recreation. |
| Dieback of Mediterranean maquis vegetation: La Maddelena archipelago, Italy | Natural vegetation | 9 ^d^ | 6, 7, 8 | Root lesions, collar lesions, stem lesions leading to mortality and decline of natural vegetation in a National Park. Impact on tourism, biodiversity and natural heritage. |
| Restoration plantings in native Mediterranean heath vegetation and woodlands:  Bay area, California | Planting, specialist nursery | 51 ^e^ | 1, 2, 4, 6,7,8 | Strong evidence for spread to native plant habitats of at least five *Phytophthora* species causing root lesions, collar lesions, dieback and mortality of the vegetation. Impact on biodiversity and natural heritage. |
| Dieback of eucalypt forests, Banksia woodlands and heath vegetation: across Western Australia | Forest, natural vegetation | 26 ^f^ |  | Root and collar rot resulting in devastating dieback of whole ecosystems. Many of the *Phytophthora* species involved are considered native; however, the most aggressive species with the widest host ranges are introduced invasives (*P. cinnamomi*, *P. elongata* and *P. multivora*). Impacts on biodiversity, conservation, forestry and natural heritage. |
| Woody plant nurseries and outplantings: across Europe | Nursery, outplantings | 65 ^g^ | 1, 2, 3, 4, 6, 7, 8, 9, 12 | Most of these *Phytophthora* taxa are not native to Europe but are now established in the wider environment causing diseases of trees and shrubs in forests and natural ecosystems (e.g. *P. austrocedri*, *P. cactorum*, *P. cinnamomi*, *P. kernoviae, P. multivora*, *P. plurivora*, *P. ramorum, P. ×alni*, *P. ×cambivora*). Direct impact on nurseries; indirect impact on forestry, private garden owners, recreation and natural heritage. |

^a^ *P. botryosa*, *P. capsici*, *P. heveae*, *P. megakarya*, *P. palmivora* (Erwin & Ribeiro 1996).

^b^ *P. cactorum, P. castaneae, P. cinnamomi, P. citricola, P. cryptogea, P. drechsleri, P. bilorbang, P. chlamydospora, P. europaea, P. gallica, P. gonapodyides, P. lacustris, P. megasperma, P. multivora, P. plurivora, P. pseudocryptogea, P. pseudosyringae, P. psychrophila, P. quercina, P. ramorum, P. syringae, P. tyrrhenica, P. uliginosa, P. ×cambivora, P.* taxon forest soil, *P.* taxon river soil (Brasier et al. 1993; Jung et al. 1996, 2000, 2013, 2017c, 2018a, 2019; Vettraino et al. 2002; Balci & Halmschlager 2003; Seddaiu et al. 2021)

^c^ *P. cactorum, P. chlamydospora, P. castaneae, P. cinnamomi, P. europaea, P. gonapodyides, P. kernoviae*, *P. plurivora, P. pseudosyringae, P. psychrophila, P. ramorum, P. syringae, P. tubulina, P. uliginosa, P. vulcanica, P. ×cambivora* (Cacciola et al. 2005; Jung et al. 2005, 2013b, 2017c, 2018a, 2019; Hartmann et al. 2006; Brown & Brasier 2007; Jung 2009; Schmitz et al. 2009; Stępniewska & Dłuszyński 2010; Telfer et al. 2015).

^d^ *P.* *asparagi, P. bilorbang, P. cinnamomi, P. crassamura, P. gonapodyides, P. melonis, P. ornamentata, P. pseudocryptogea, P. syringae* (Scanu et al. 2015).

^e^ *P. acerina, P. amnicola, P. bilorbang, P. borealis, P. cactorum*, *P. chlamydospora*, *P. citricola*, *P. crassamura*, *P. cryptogea*, *P. drechsleri*, *P. erythroseptica*, *P. europaea*, *P. gonapodyides*, *P. hedraiandra*, *P. inundata*, *P. lacustris*, *P. megasperma*, *P. nicotianae*, *P. multivora*, *P. niederhauserii*, *P. kelmanii,* *P. occultans*, *P. palmivora*, *P. parvispora*, *P. plurivora*, *P. pseudocryptogea*, *P. quercetorum*, *P. ramorum*, *P. riparia*, *P. rosacearum*, *P. siskiyouensis*, *P. tentaculata*, *P. thermophila, P. tropicalis, P.* ×*cambivora, P.* aff. *citricola, P.* aff. *lacustris, P.* taxon agrifolia, *P.* taxon “cactorum-like”, *P*. taxon citricola-pini complex, *P*. taxon kelmania type-2, *P*. taxon niederhauserii-like, *P*. taxon raspberry, eight undescribed *Phytophthora* hybrids (Garbelotto et al. 2018; Frankel et al. 2020).

^f^ *P. amnicola*, *P. arenaria*, *P. balyanboodja*, *P. boodjera*, *P. cinnamomi*, *P. condilina*, *P. cooljarloo*, *P. constricta*, *P. crassamura*, *P. cryptogea*, *P. elongata*, *P. gibbosa*, *P. gregata*, *P. humicola*, *P. inundata*, *P. kelmanii*, *P. kwongonina*, *P. litoralis*, *P. moyootj*, *P. multivora*, *P. personensis*, *P. pseudocryptogea*, *P. pseudorosacearum*, *P. rosacearum*, *P. thermophila*, *P. versiformis* (Shearer & Tippett 1989; Shearer et al. 2004; Scott et al. 2009; Rea et al. 2010, 2011; Jung et al. 2011, 2013; Paap et al. 2017; Belhaj et al. 2018; Burgess et al. 2018).

^g^ *P. asparagi*, *P. austrocedrae*, *P. bilorbang*, *P. cactorum*, *P. capensis*, *P. capsici*, *P. castanetorum*, *P. chlamydospora*, *P. chrysanthemi*, *P. cinnamomi*, *P. citrophthora*, *P. citricola*, *P. clandestina*, *P. cryptogea*, *P. drechsleri*, *P. foliorum, P. fragariae*, *P. gonapodyides*, *P. hedraiandra*, *P. heterospora*, *P. hibernalis*, *P. humicola*, *P. hydropathica*, *P. ilicis*, *P. infestans*, *P. inundata*, *P. kelmanii*, *P. lacustris*, *P. lateralis*, *P. megasperma*, *P. melonis*, *P. multivora*, *P. nemorosa*, *P. nicotianae*, *P. niederhauserii*, *P. pachypleura*, *P. palmivora*, *P. parvispora*, *P. uniformis*, *P. pini*, *P. plurivora*, *P. pseudocryptogea*, *P. pseudosyringae*, *P. pseudotsugae*, *P. psychrophila*, *P. quercina*, *P. quercetorum*, *P. ramorum*, *P. richardiae*, *P. rubi*, *P. sojae*, *P. syringae*, *P. tentaculata*, *P. transitoria*, *P. tropicalis*, *P. tubulina*, *P. uliginosa*, *P.* ×*alni*, *P.* ×*cambivora*, *P.* ×*pelgrandis*, *P.* ×*serendipita*, *P. citricola* 5 , *P. citricola* 6, *P.* taxon ‘paludosa’, *P.* taxon ‘pseudocitricola’, *P.* taxon ‘raspberry’, *P.* taxon ‘walnut’ (Moralejo et al. 2009; Man In' t Veld et al. 2012; Jung et al. 2016; Rossmann et al. 2021; Scanu et al. 2021).

References

Balci Y, Halmschlager E (2003) Incidence of *Phytophthora* species in oak forests in Austria and their possible involvement in oak decline. *Forest Pathol* 33:157–174.

Belhaj R, McComb J, Burgess TI, Hardy GEStJ (2018) Pathogenicity of 21 newly described *Phytophthora* species against seven Western Australian native plant species. *Plant Pathol* 67:1140–1149.

Brasier CM, Robredo F, Ferraz JFP (1993) Evidence for *Phytophthora cinnamomi* involvement in Iberian oak decline. Plant Pathol 42:140–145. <https://doi.org/10.1111/j.1365-3059.1993.tb01482.x>

Brown AV, Brasier CM (2007) Colonization of tree xylem by *Phytophthora ramorum*, *P. kernoviae* and other *Phytophthora* species. Plant Pathol 56:227–241. <https://doi.org/10.1111/j.1365-3059.2006.01511.x>

Burgess TI, Simamora AV, White D, Wiliams B, Schwager M, Stukely MJC, Hardy GEStJ (2018) New species from *Phytophthora* Clade 6a: evidence for recent radiation. Persoonia 41: 1–7. <https://doi.org/10.3767/persoonia.2018.41.01>

Cacciola SO, Diana G, Pane A, Chimento A, Raudino F (2005) Un focolaio di cancro basale gommoso del faggio causato da *Phytophthora pseudosyringae* nel Parco Nazionale d’Abruzzo. *Informatore Fitopatologico* 55:52–58.

Erwin DC, Ribeiro OK (1996) Phytophthora diseases worldwide. American Phytopathological Society (APS Press), St. Paul, Minnesota:562 pp.

Frankel SJ, Conforti C, Hillman J, Ingolia M, Shor A, Benner D, Alexander JM, Bernhardt E, Swiecki TJ (2020) *Phytophthora* introductions in restoration areas: Responding to protect California native flora from human-assisted pathogen spread. Forests 11:1291.

Garbelotto M, Frankel SJ, Scanu B (2018) Soil- and waterborne *Phytophthora* species linked to recent outbreaks in Northern California restoration sites. *California Agriculture* 72:208–216.

Jung T (2009) Beech decline in Central Europe driven by the interaction between Phytophthora infections and climatic extremes. Forest Pathol 39:73–94. <https://doi.org/10.1111/j.1439-0329.2008.00566.x>

Jung T, Blaschke H, Neumann P (1996) Isolation, identification and pathogenicity of *Phytophthora* species from declining oak stands. Eur J For Pathol 26:253–272.

Jung T, Horta Jung M, Cacciola SO, Cech T, Bakonyi J, Seress D, Mosca S, Schena L, Seddaiu S, Pane A, Magnano di San Lio G, Maia C, Cravador C, Franceschini A, Scanu B (2017c) Multiple new cryptic pathogenic *Phytophthora* species from Fagaceae forests in Austria, Italy and Portugal. IMA Fungus 8:219–244. <https://doi.org/10.5598/imafungus.2017.08.02.02>

Jung T, Hudler GW, Jensen-Tracy SL, Griffiths HM, Fleischmann F, Oßwald W (2005) Involvement of *Phytophthora* spp. in the decline of European beech in Europe and the USA. Mycologist 19:159–166.

Jung T, La Spada F, Pane A, Aloi F, Evoli M, Horta Jung M, Scanu B, Faedda R, Rizza C, Puglisi I, di San Magnano, Lio G (2019) Diversity and distribution of *Phytophthora* species in protected natural areas in Sicily. Forests 10:259.

Jung T, Orlikowski L, Henricot B, Abad-Campos P, Aday AG, Aguín Casal O, Bakonyi J, Cacciola SO, Cech T, Chavarriaga D, Corcobado T, Cravador A, Decourcelle T, Denton G, Diamandis S, Doğmuş-Lehtijärvi HT, Franceschini A, Ginetti B, Green S, Glavendekić M, Hantula J, Hartmann G, Herrero M, Ivic D, Horta Jung M, Lilja A, Keca N, Kramarets V, Lyubenova A, Machado H, Magnano di San Lio G, Mansilla Vázquez PJ, Marçais B, Matsiakh I, Milenkovic I, Moricca S, Nagy ZÁ, Nechwatal J, Olsson C, Oszako T, Pane A, Paplomatas EJ, Pintos Varela C, Prospero S, Rial Martínez C, Rigling D, Robin C, Rytkönen A, Sánchez ME, Sanz Ros AV, Scanu B, Schlenzig A, Schumacher J, Slavov S, Solla A, Sousa E, Stenlid J, Talgø V, Tomic Z, Tsopelas P, Vannini A, Vettraino AM, Wenneker M, Woodward S, Peréz-Sierra A (2016) Widespread *Phytophthora* infestations in European nurseries put forest, semi-natural and horticultural ecosystems at high risk of Phytophthora diseases. Forest Pathol 46:134–163. <https://doi.org/10.1111/efp.12239>

Jung T, Pérez-Sierra A, Durán A, Jung MH, Balci Y, Scanu B (2018a) Canker and decline diseases caused by soil- and airborne *Phytophthora* species in forests and woodlands. Persoonia 40:182–220. <https://doi.org/10.3767/persoonia.2018.40.08>

Jung T, Stukely MJC, Hardy GEStJ, White D, Paap T, Dunstan WA, Burgess TI (2011) Multiple new *Phytophthora* species from ITS Clade 6 associated with natural ecosystems in Australia: evolutionary and ecological implications. Persoonia 26:13–39. <https://doi.org/10.3767/003158511X557577>

Jung T, Vettraino AM, Cech TL, Vannini A (2013b) The impact of invasive *Phytophthora* species on European forests. In: Lamour K (ed), Phytophthora: A global perspective: CABI, Wallingford, UK:146–158.

Man in ‘t Veld WA, Rosendahl KCHM, Hong C (2012) *Phytophthora* ×*serendipita* sp. nov. and *P*. ×*pelgrandis*, two destructive pathogens generated by natural hybridization. Mycologia 104:1390–1396. <https://doi.org/10.3852/11-272>

Moralejo E, Pérez-Sierra A, Alvarez LA, Belbahri L, Lefort F, Descals E (2009) Multiple alien *Phytophthora* taxa discovered on diseased ornamental plants in Spain. Plant Pathol 58:100–110. <https://doi.org/10.1111/j.1365-3059.2008.01930.x>

Paap T, Croeser L, White D, Aghighi S, Barber P, Hardy GEStJ, Burgess TI (2017) *Phytophthora versiformis* sp. nov., a new species from Australia related to *P. quercina*. Australas Plant Path 46:369–378 DOI 10.1007/s13313-017-0499-7

Rea AJ, Jung T, Burgess TI, Stukely MJC, Hardy GEStJ (2010) *Phytophthora elongata* sp. nov. a novel pathogen from the *Eucalyptus marginata* forest of Western Australia. Australas Plant Pathol 39:477–491.

Rea AJ, Burgess TI, Hardy GEStJ, Stukely MJC, Jung T (2011) Two novel and potentially endemic species of *Phytophthora* associated with episodic dieback of kwongan vegetation in the south-west of Western Australia. Plant Pathol 60:1055–1068. <https://doi.org/10.1111/j.1365-3059.2011.02463.x>

Rossmann S, Lysøe E, Skogen M, Talgø V, Brurberg MB (2021) DNA metabarcoding reveals broad presence of plant pathogenic oomycetes in soil from internationally traded plants. Front Microbiol 12:637068.

Scanu B, Linaldeddu BT, Deidda A, Jung T (2015) Diversity of *Phytophthora* species from declining Mediterranean maquis vegetation, including two new species, *Phytophthora crassamura* and *P. ornamentata* sp. nov. PLoS One 10:e0143234. <https://doi.org/10.1371/journal.pone.0143234>

Scanu B, Jung T, Masigol H, Linaldeddu BT, Horta Jung M, Brandano A, Mostowfizadeh-Ghalamfarsa R, Janoušek J, Riolo R, Cacciola SO (2021) *Phytophthora heterospora* sp. nov., a new pseudoconidia-producing sister species of *P. palmivora*. J. Fungi 7:870. <https://doi.org/10.3390/jof7100870>

Schmitz S, Zini J, Chandelier A (2009) Involvement of *Phytophthora* species in the decline of beech (*Fagus sylvatica*) in the southern part of Belgium. In: Goheen EM, Frankel SJ (eds), Phytophthoras in Forests and Natural Ecosystems: Fourth Meeting of the International Union of Forest Research Organizations (IUFRO) Working Party S07.02.09, General Technical Report PSW-GTR-221, USDA Forest Service, Pacific Southwest Research Station, Albany, California:320–323.

Scott PM, Burgess TI, Barber PA, Shearer BL, Stukely MJC, Hardy, Jung, T (2009) *Phytophthora multivora* sp. nov., a new species recovered from declining *Eucalyptus*, *Banksia*, *Agonis* and other plant species in Western Australia. Persoonia 22:1–13.

Seddaiu S, Brandano A, Ruiu PA, Sechi C, Scanu B (2020). An overview of *Phytophthora* species inhabiting declining *Quercus suber* stands in Sardinia (Italy). Forests 11:971.

Shearer BL, Crane CE, Cochrane A (2004) Quantification of the susceptibility of the native flora of the South-West Botanical Province, Western Australia, to *Phytophthora cinnamomi*. Aust J Bot 52:435–443. <https://doi.org/10.1071/BT03131>

Shearer BL, Tippett JT (1989) Jarrah dieback: The dynamics and manage- ment of *Phytophthora cinnamomi* in the jarrah (*Eucalyptus marginata*) forests of south-western Australia. Perth, Department of Conservation and Land Management.

Stępniewska H, Dłuszyński J (2010) Incidence of *Phytophthora* *cambivora* in bleeding lesions on beech stems in selected forest stands in south-eastern Poland. Phytopathologia 56:39–51.

Telfer KH, Brurberg MB, Herrero ML, Stensvand A, Talgø V (2015) *Phytophthora* *cambivora* found on beech in Norway. Forest Pathol 45:415–425.

Vettraino AM, Barzanti GP, Bianco MC, Ragazzi A, Capretti P, Paoletti E, Luisi N, Anselmi N, Vannini A (2002) Occurrence of *Phytophthora* species in oak stands in Italy and their association with declining oak trees. Forest Pathol 32:19–28.
