# Supplementary material for: Phytophthora: an ancient, historic, biologically and structurally cohesive and evolutionarily successful generic concept in need of preservation
Source: IMA Fungus. 2022 Jun 27;13:12. doi: 10.1186/s43008-022-00097-z (PMC9235178; doi:10.1186/s43008-022-00097-z)
Supplement: Supplementary file 3 — Additional file 3: Table S3. Main morphological characters and breeding systems of 196 culturable Phytophthora species in the different clades (number/percentage of species per clade). [file 43008_2022_97_MOESM3_ESM.docx]

**Table S3:** Main morphological characters and breeding systems of 196 culturable *Phytophthora* species in the different clades (number / percentage of species per clade).^a^

| **Clade**  **(no. of species)** | **Sporangial apex** | | | **Caducity** | | **Breeding system** | | | **Antheridial insertion^b^** | | | **Chlamydospores** | |
| --- | --- | --- | --- | --- | --- | --- | --- | --- | --- | --- | --- | --- | --- |
|  | **papillate** | **semi-papillate** | **non-papillate** | **caducous** | **persistent** | **homo-thallic** | **hetero-**  **thallic** | **sterile** | **amphi-gynous** | **para-**  **gynous** | **amphi- & para-**  **gynous** | **present** | **absent** |
| **1** (19) | 12 / **63.2** | 7 / **36.8** |  | 13 / **68.4** | 6 / **31.6** | 11 / **57.9** | 8 / **42.1** |  | 9 / **47.4** | 8 / **42.1** | 2 / **10.5** | 4 / **21.1** | 15 / **78.9** |
| **2** (36) | 18 / **50.0** | 17 / **47.2** | 1 / **2.8** | 14 / **38.9** | 22 / **61.1** | 22 / **61.1** | 12 / **33.3** | 2 / **5.6** | 15 / **44.1** | 17 / **50.0** | 2 / **5.9** | 10 / **27.8** | 26 / **72.2** |
| **3** (6) |  | 5 / **83.3** | 1 / **16.7** | 5 / **83.3** | 1 / **16.7** | 5 / **83.3** |  | 1 / **16.7** | 4 / **80.0** | 1 / **20.0** |  | 1 / **16.7** | 5 / **83.3** |
| **4** (10) | 10 / **100** |  |  | 4 / **40.0** | 6 / **60.0** | 7 / **70.0** | 3 / **30.0** |  | 4 / **40.0** | 6 / **60.0** |  | 3 / **30.0** | 7 / **70.0** |
| **5** (4) | 4 / **100** |  |  |  | 4 / **100** | 4 / **100** |  |  | 4 / **100** |  |  |  | 4 / **100** |
| **6** (33) |  |  | 33 / **100** | 1 / **3.0** | 32 / **97.0** | 15 / **45.5** | 3 / **9.1** | 15 / **45.5** | 8 / **44.4** | 10 / **55.6** |  | 9 / **27.3** | 24 / **72.7** |
| **7** (31) |  |  | 31 / **100** | 1 / **3.2** | 31 / **100** | 23 / **74.2** | 8 / **25.8** |  | 16 / **51.6** | 11 / **35.5** | 4 / **12.9** | 7 / **22.6** | 24 / **77.4** |
| **8** (25) |  | 13 / **52.0** | 12 / **48.0** | 5 / **20.0** | 20 / **80.0** | 18 / **72.0** | 7 / **28.0** |  | 12 / **48.0** | 11 / **44.0** | 2 / **8.0** | 7 / **28.0** | 18 / **72.0** |
| **9** (20) |  |  | 20 / **100** | 3 / **15.0** | 17 / **85.0** | 9 / **45.0** | 6 / **30.0** | 5 / **25.0** | 9 / **60.0** | 4 / **26.7** | 1^c^ / **6.7** | 11 / **55.0** | 9 / **45.0** |
| **10** (7) | 4 / **57.1** |  | 3 / **42.9** | 4 / **57.1** | 3 / **42.9** | 4 / **57.1** | 1 / **14.3** | 2 / **28.6** | 5 / **100** |  |  | 4 / **57.1** | 3 / **42.9** |
| **11** (1) |  |  | 1 / **100** |  | 1 / **100** | 1 / **100** |  |  |  | 1 / **100** |  | 1 / **100** |  |
| **12** (4) | 4 / **100** |  |  |  | 4 / **100** | 4 / **100** |  |  |  | 4 / **100** |  | 2 / **50.0** | 2 / **50.0** |
| **No. / % of Clades^d^** | 6 / **54.5** | 4 / **36.4** | 7 / **63.6** | 9 / **81.8** | 11 / **100** | 11 / **100** | 8 / **72.7** | 5 / **45.5** | 10 / **90.9** | 8 / **72.7** | 5 / **45.5** | 10 / **90.9** | 11 / **100** |
| **No. / % of species** | 52 / **26.5** | 42 / **21.4** | 102 / **52.0** | 50 / **25.5** | 146 / **74.5** | 123 / **62.8** | 48 / **24.5** | 25 / **12.7** | 86 / **50.3** | 73 / **42.7** | 11 / **6.4** | 59 / **30.1** | 137 / **69.9** |

^a^ Data sourced from Erwin & Ribeiro (1996), from the respective species descriptions and from morphological studies performed by the authors.

^b^ Proportions of antheridial insertion types calculated only for the 170 sexual species which produce antheria.

^c^ *P. insolita* forms oogonia without antheridia.

^d^ Numbers and proportions of clades calculated without Clade 11 which included only 1 species.
