# Supplementary material for: Phytophthora: an ancient, historic, biologically and structurally cohesive and evolutionarily successful generic concept in need of preservation
Source: IMA Fungus. 2022 Jun 27;13:12. doi: 10.1186/s43008-022-00097-z (PMC9235178; doi:10.1186/s43008-022-00097-z)
Supplement: Supplementary file 4 — Additional file 4: Table S4. Lifestyles, diseases and host ranges of 196 culturable Phytophthora species in the different clades (number/percentage of species per clade). [file 43008_2022_97_MOESM4_ESM.docx]

**Table S4:** Lifestyles, diseases and host ranges of 196 culturable *Phytophthora* species in the different clades (number / percentage of species per clade).^a^

| **Clade**  **(no. of species)** | **Lifestyles** | | | | **Known diseases** | | | | **Known host ranges** | | | | | **Sapro-trophic in water bodies** |
| --- | --- | --- | --- | --- | --- | --- | --- | --- | --- | --- | --- | --- | --- | --- |
|  | **Soil-borne** | **Aerial & soil-borne** | **Aerial** | **Aquatic** | **Root rot** | **Stem cankers on woody hosts** | **Leaf/ shoot blight, bud rot, fruit rot** | **Decline/dieback of forest eco-systems** | **Woody host plants** | **Wide; >20 hosts** | **Medium; 6-20 hosts** | **Narrow; up to 5 hosts** | **Host-specific (species, genus)** |  |
| **1** (19) | 7 / **36.8** | 5 / **26.3** | 7 / **36.8** |  | 12/ **63.2** | 7 / **36.8** | 10 / **52.6** | 3 / **15.8** | 10 / **52.6** | 3 / **15.8** | 4 / **21.1** | 9 / **47.4** | 9 / **47.4** | 3 / **15.8** |
| **2** (36) | 23 / **63.9** | 9/ **25.0** | 4 / **11.1** |  | 26 / **72.2** | 19 / **52.7** | 14 / **38.9** | 9 / **25.0** | 32 / **88.9** | 5 / **13.9** | 7 / **19.4** | 24 / **66.7** | 1 / **2.8** | 14 / **38.9** |
| **3** (6) | 1 / **16.7** | 2 / **33.3** | 3 / **50.0** |  | 3 / **50.0** | 3 / **50.0** | 4 / **66.7** | 4 / **66.7** | 6 / **100** |  | 3 / **50.0** | 3 / **50.0** | 1 / **16.7** | 3 / **50.0** |
| **4** (10) | 6 / **60** | 2 / **20.0** | 2 / **20.0** |  | 8 / **80.0** | 4 / **40.0** | 3 / **30.0** | 4 / **40.0** | 10 / **100** | 1 / **10.0** | 5 / **50.0** | 3 / **30.0** | 2 / **20.0** | 2 / **20.0** |
| **5** (4) | 4 / **100** |  |  |  | 3 / **75.0** | 3 / **75.0** | 2 / **50.0** | 2 / **50.0** | 4 / **100** | 2 / **50.0** |  | 2 / **50.0** | 2 ?/ **50?** | 2 / **50.0** |
| **6** (33) | 24 / **72.7** |  | 1 / **3.0** | 22 / **66.7** | 24 / **72.7** | 5 / **15.2** | 1 / **3.0** | 6 / **18.2** | 24 / **72.7** | 1 / **3.0** | 8 / **24.2** | 18 / **54.5** | 1 / **3.0** | 25 / **75.8** |
| **7** (31) | 31 / **100** |  |  | 2 / **6.5** | 31 / **100** | 10 / **32.3** | 6 / **19.4** | 9 / **29.0** | 23 / **74.2** | 4 / **12.9** | 3 / **9.7** | 20 / **64.5** | 5 / **16.1** | 10 / **32.3** |
| **8** (25) | 20 / **80.0** | 1 / **4.0** | 3 / **12.0** | 2 / **8.0** | 23 / **92.0** | 6 / **24.0** | 6 / **24.0** | 4 / **16.0** | 12 / **48.0** | 3 / **12.0** | 5 / **20.0** | 16 / **64.0** | 10 / **40.0** | 9 / **36.0** |
| **9** (20) | 9 / **45.0** | 2 / **10.0** |  | 13 / **65** | 8 / **40.0** | 2 / **10.0** | 3 / **15.0** | 4 / **20.0** | 10 / **50.0** |  | 3 / **15.0** | 9 / **45.0** |  | 14 / **70.0** |
| **10** (7) | 2 / **28.6** | 2 / **28.6** | 2 / **28.6** | 2 / **28.6** | 5 / **71.4** | 1 / **14.3** | 3 / **42.9** | 1 / **14.3** | 6 / **85.7** | 1 / **14.3** | 1 / **14.3** | 4 / **57.1** |  | 3 / **42.9** |
| **11** (1) | 1 / **100** |  |  |  | 1 / **100** |  |  |  |  |  |  | 1 / **100** |  |  |
| **12** (4) | 4 / **100** |  |  |  | 4 / **100** |  |  | 1 / **25.0** | 4 / **100** |  | 2 / **50.0** | 2 / **50.0** | 3 / **75.0** |  |
| **No. / % of Clades^a^** | 11 / **100** | 7 / **63.6** | 7 / **63.6** | 5 / **45.5** | 11 / **100** | 10 / **90.9** | 10 / **90.9** | 11 / **100** | 11 / **100** | 8 / **72.7** | 10 / **90.9** | 11 / **100** | 9 / **81.8** | 10 / **90.9** |
| **No. / % of species** | 132 / **67.3** | 23 / **11.7** | 22 / **11.2** | 41 / **20.9** | 148 / **75.5** | 60 / **30.6** | 52 / **26.5** | 47 / **24.0** | 141 / **71.9** | 20 / **10.2** | 40 / **20.4** | 102 / **52.0** | 35 / **17.9** | 85 / **43.4** |

^a^ Data sourced from Erwin & Ribeiro (1996), the respective species descriptions and extensive phytopathological literature.

^b^ Number and proportion of clades calculated without Clade 11 which included only 1 species.
