# Supplementary material for: Phytophthora: an ancient, historic, biologically and structurally cohesive and evolutionarily successful generic concept in need of preservation
Source: IMA Fungus. 2022 Jun 27;13:12. doi: 10.1186/s43008-022-00097-z (PMC9235178; doi:10.1186/s43008-022-00097-z)
Supplement: Supplementary file 5 — Additional file 5: Table S5. Optimum and maximum temperatures for growth of 196 culturable Phytophthora species in the different clades (number/percentage of species per clade). [file 43008_2022_97_MOESM5_ESM.docx]

**Table S5:** Optimum and maximum temperatures for growth of 196 culturable species in the different *Phytophthora* clades (number / percentage of species per clade).^a^

| **Clade**  **(no. of species)** | **Optimum temperature** | | | **Maximum temperature** | | | | **Cardinal temperatures unknown** |
| --- | --- | --- | --- | --- | --- | --- | --- | --- |
|  | **low**  **≤ 20°C** | **medium**  **>20-27.5°C** | **high**  **> 27.5°C** | **low**  **≤ 25°C** | **medium**  **>25-30°C** | **high**  **>30°-<35°C** | **very high**  **≥35°C** |  |
| **1** (19) | 10 / **52.6** | 7 / **36.8** | 2 / **10.5** | 2 / **10.5** | 9 / **47.4** | 6 / **31.6** | 2 / **10.5** |  |
| **2** (36) | 3 / **8.6** | 26 / **74.3** | 6 / **17.1** |  | 8 / **22.9** | 22 / **62.9** | 5 / **14.3** | 1 |
| **3** (6) | 5 / **83.3** | 1 / **16.7** |  | 4 / **66.7** | 2 / **33.3** |  |  |  |
| **4** (10) |  | 7 / **70.0** | 3 / **30.0** |  | 2 / **20.0** | 7 / **70.0** | 1 / **10.0** |  |
| **5** (4) |  | 4 / **100** |  | 1 / **25.0** | 2 / **50.0** | 1 / **25.0** |  |  |
| **6** (33) | 2 / **6.1** | 13 / **39.4** | 18 / **54.5** |  | 3 / **9.1** | 12 / **36.4** | 18 / **54.5** |  |
| **7** (31) | 5 / **17.2** | 19 / **65.5** | 5 / **17.2** | 2 / **6.9** | 3 / **10.3** | 18 / **62.1** | 6 / **20.7** | 2 |
| **8** (25) | 13 / **52.0** | 10 / **40.0** | 2 / **8.0** | 6 / **24.0** | 10 / **40.0** | 6 / **24.0** | 3 / **12.0** |  |
| **9** (20) | 2 / **10.0** | 3 / **15.0** | 15 / **75.0** | 1 / **5.0** | 3 / **15.0** | 3 / **15.0** | 13 / **65.0** |  |
| **10** (7) | 3 / **42.9** | 4 / **57.1** |  | 1 / **14.3** | 2 / **28.6** | 4 / **57.1** |  |  |
| **11** (1) |  |  | 1 / **100** |  |  | 1 / **100** |  |  |
| **12** (4) | 3 / **75.0** | 1 / **25.0** |  |  | 4 / **100** |  |  |  |
| **No. / % of Clades^b^** | 9 / **81.1** | 11 / **100** | 7 / **63.6** | 7 / **63.6** | 11 / **100** | 9 / **81.1** | 7 / **63.6** | n.a. |
| **No. / % of species^c^** | 46 / **23.8** | 95 / **49.2** | 52 / **27.0** | 17 / **8.8** | 48 / **24.9** | 80 / **41.5** | 48 / **24.9** | n.a. |

^a^ Data sourced from Erwin & Ribeiro (1996), the respective species descriptions and temperature-growth tests performed by the authors.

^b^ Numbers and proportions of clades calculated without Clade 11 which included only 1 species.

^c^ Proportions of species calculated only for the 193 species with known cardinal temperatures for growth.
