# Supplementary material for: Phytophthora: an ancient, historic, biologically and structurally cohesive and evolutionarily successful generic concept in need of preservation
Source: IMA Fungus. 2022 Jun 27;13:12. doi: 10.1186/s43008-022-00097-z (PMC9235178; doi:10.1186/s43008-022-00097-z)
Supplement: Supplementary file 6 — Additional file 6: Table S6. Sporangial characteristics of 164 species within Phytophthora Clades 1–5 and Clades 6–8. [file 43008_2022_97_MOESM6_ESM.docx]

**Table S6.** Sporangial characteristics of 164 species within *Phytophthora* Clades 1-5 and Clades 6-8

|  | **Percent (no.) of species with sporangial type** | | | | | |
| --- | --- | --- | --- | --- | --- | --- |
|  | **Papillate caducous** | **Semipapillate fully or partially caducous** | **Nonpapillate fully or partially caducous** | **Papillate persistent** | **Semipapillate persistent** | **Nonpapillate persistent** |
| Clades 1-5 (75 species) | 30.7 (23) | 18.7 (14) | 0 (0) | 32.0 (24) | 16.0 (12) | 2.7 (2) |
| Clades 6-8 (89 species) | 0 (0) | 11.2 (10) | 3.4 (3) | 0 (0) | 4.5 (4) | 80.9 (72) |
