# Supplementary material for: Phytophthora: an ancient, historic, biologically and structurally cohesive and evolutionarily successful generic concept in need of preservation
Source: IMA Fungus. 2022 Jun 27;13:12. doi: 10.1186/s43008-022-00097-z (PMC9235178; doi:10.1186/s43008-022-00097-z)
Supplement: Supplementary file 7 — Additional file 7: Table S7. Unusual morphological or developmental features among Phytophthora species. [file 43008_2022_97_MOESM7_ESM.docx]

**Table S7.** Unusual morphological or developmental features among *Phytophthora* species

| **Species** | **Clade** | **Features** | **Possible adaptations** |
| --- | --- | --- | --- |
| *P. infestans* | 1 | Sporangiophore apophyses ^a^ | Mechanism of indeterminate sporangiophore growth |
| *P. capsici* | 2 | Long pedicels 30->100 µm ^b^  Umbellate sympodia ^c^ | Splash dispersal, sporangial clustering and adherence to host surfaces. |
| *P. litchii* | 4 | Downy white mycelium ^d^  Determinate sporangiophores and synchronous sporangial formation ^d^ | Resistance to dessication on suberised fruit pericarp surface  Rapid synchronized sporulation on exposed fruit pericarp surface |
| *P. heterospora* | 4 | Pseudoconidia: direct germination, no papillum; formed alongside papillate zoosporic sporangia ^e^ | Adaptation to both moist and drier habitats or seasonal or diurnal climate |
| *P. pinifolia* | 6 | Narrowing of sporangiophores near sporangial bases ^f^ | Facultative caducity, enabling aerial infection and dispersal in a Clade of soil- and water-borne species |
| *P. cinnamomi* | 7 | Tough mycelium ^g^  Stromata ^h^  Lignitubers (intracellular hyphae encased in callose layers produced by the host cell) ^h^ | Competitive growth through soil and litter  Nutrient storage for seasonal hibernation and subsequent sporulation  Long term survival |
| *P. lateralis* | 8 | Stromata and sporangiomata ^i^ | Pressure eruption through needle cuticle followed by sporulation |
| *P. hibernalis* | 8 | Long pedicels 20–80 µm ^c^ | Splash dispersal, sporangial clustering and adherence to host surfaces. |
| *P. ramorum* | 8 | Stromata and sporangiomata ^j^ | Pressure eruption through tough leaf cuticle or fruit periderm followed by sporulation |
| *P. constricta* | 9 | Sporangiophore constrictions near the sporangial bases ^k^ | Facultative caducity and aerial dispersal in a Clade of soil- and waterborne species |
| *P. insolita* | 9 | Production of oospores without antheridia (presumed gametangial apomixis) ^l^ | Inbreeding mechanism. Survival in periodically dry waterways without cost of less adapted recombinant offspring |

References

^a^ Thines M (2009) Bridging the gulf: *Phytophthora* and downy mildews are connected by rare grass parasites. PloS One 4:e4790. <https://doi.org/10.1371/journal.pone.0004790>

^b^ Kunimoto RK, Aragaki M, Hunter JE, Ko WH (1976) *Phytophthora capsici*, corrected name for the cause of Phytophthora blight of Macadamia racemes. Phytopathology 66:546–548. <https://doi.org/10.1094/Phyto-66-546>.

^c^ Erwin DC, Bartnicki-Garcia S, Tsao PH (eds) (1983) *Phytophthora*: Its Biology, Taxonomy, Ecology and Pathology. American Phytopathological Society, St. Paul, Minnesota:392 pp.

^d^ Chen CC (1961) A species of *Peronophythora* gen. nov. parasitic on litchi fruit in Taiwan. National Taiwan University, Chinese Taipei, Special Publication of College of Agriculture 10:37 pp.

Ho HH, Lu JY, Gong LY (1984) Observations on sexual reproduction by *Peronophythora litchii*. Mycologia 76:745–747. <https://doi.org/10.1080/00275514.1984.12023906>

Sun J, Gao Z, Zhang X, Zou X, Cao L, Wang J (2017) Transcriptome analysis of *Phytophthora litchii* reveals pathogenicity arsenals and confirms taxonomic status. Plos One 12(6):e0178245. <https://doi.org/10.1371/journal.pone.0178245>

Ye W, Wang Y, Shen D, Li D, Pu T, Jiang Z, Zhang Z, Zheng X, Tyler BM, Wang Y (2016) Sequencing of the litchi downy blight pathogen reveals it is a *Phytophthora* species with downy mildew-like characteristics. Mol Plant Microbe In 29(7):573–583. <https://doi.org/10.1094/MPMI-03-16-0056-R>

^e^ Scanu B, Jung T, Masigol H, Linaldeddu BT, Horta Jung M, Brandano A, Mostowfizadeh-Ghalamfarsa R, Janoušek J, Riolo R, Cacciola SO (2021) *Phytophthora heterospora* sp. nov., a new pseudoconidia-producing sister species of *P. palmivora*. J. Fungi 7:870. <https://doi.org/10.3390/jof7100870>

^f^ Durán A, Gryzenhout M, Slippers B, Ahumada R, Rotella A, Flores F, Wingfield BD, Wingfield MJ (2008) *Phytophthora pinifolia* sp. nov. associated with a serious needle disease of *Pinus radiata* in Chile. Plant Pathol 57:715–727.

^g^ Zentmyer GA, Mircetich SM (1966) Saprophytism and persistence in soil by *Phytophthora* *cinnamomi*. Phytopathology 56:710–712.

Weste G (1983) Population dynamics and survival of *Phytophthora*. In *Phytophthora*. Its Biology, Taxonomy, Ecology, and Pathology (Erwin, D.C., Bartnicki-Garcia, S. and Tsao, P., eds), St Paul, MN: American Phytopathological Society:237–257.

^h^ Crone M, McComb JA, O’Brien PA, Hardy GEStJ (2013) Survival of *Phytophthora cinnamomi* as oospores, stromata, and thick-walled chlamydospores in roots of symptomatic and asymptomatic annual and herbaceous perennial plant species. Fungal Biol 117:112–123.

Jung T, Colquhoun IJ, Hardy GEStJ. 2013. New insights into the survival strategy of the invasive soilborne pathogen *Phytophthora cinnamomi* in different natural ecosystems in Western Australia. Forest Pathol 43:266–288. <https://doi.org/10.1111/efp.12025>

^i^ Brasier CM, Vettraino AM, Chang TT, Vannini A (2010) *Phytophthora lateralis* discovered in an old growth *Chamaecyparis* forest in Taiwan. Plant Pathol 59:595–603. <https://doi.org/10.1111/j.1365-3059.2010.02278.x>

^j^ Moralejo E, Puig M, García JA, Descals E (2006) Stromata, sporangiomata and chlamydosori of *Phytophthora ramorum* on inoculated Mediterranean woody plants. Mycol Res 110:1323–1332. <https://doi.org/10.1016/j.mycres.2006.09.004>

^k^ Rea AJ, Burgess TI, Hardy GEStJ, Stukely MJC, Jung T (2011) Two novel and potentially endemic species of *Phytophthora* associated with episodic dieback of kwongan vegetation in the south-west of Western Australia. Plant Pathol 60:1055–1068. <https://doi.org/10.1111/j.1365-3059.2011.02463.x>

^l^ Ann PJ, Ko WH (1980) *Phytophthora insolita*, a new species from Taiwan. Mycologia 72:1180–1185.

Jung T, Stukely MJC, Hardy GEStJ, White D, Paap T, Dunstan WA, Burgess TI (2011) Multiple new *Phytophthora* species from ITS Clade 6 associated with natural ecosystems in Australia: evolutionary and ecological implications. Persoonia 26:13–39. <https://doi.org/10.3767/003158511X557577>
